# The effects of lasers on bond strength to ceramic materials: A systematic review and meta-analysis

**DOI:** 10.1371/journal.pone.0190736

**Published:** 2018-01-02

**Authors:** Verónica García-Sanz, Vanessa Paredes-Gallardo, Omel Mendoza-Yero, Miguel Carbonell-Leal, Alberto Albaladejo, José María Montiel-Company, Carlos Bellot-Arcís

**Affiliations:** 1 Department of Stomatology, Faculty of Medicine and Dentistry, University of Valencia, Valencia, Spain; 2 GROC•UJI, Institute of New Imaging Technologies, Universitat Jaume I, Castellón, Spain; 3 Department of Surgery, Faculty of Medicine, University of Salamanca, Salamanca, Spain; Federal University of Pelotas, BRAZIL

## Abstract

Lasers have recently been introduced as an alternative means of conditioning dental ceramic surfaces in order to enhance their adhesive strength to cements and other materials. The present systematic review and meta-analysis aimed to review and quantitatively analyze the available literature in order to determine which bond protocols and laser types are the most effective.

A search was conducted in the Pubmed, Embase and Scopus databases for papers published up to April 2017. PRISMA guidelines for systematic review and meta-analysis were followed.

Fifty-two papers were eligible for inclusion in the review. Twenty-five studies were synthesized quantitatively. Lasers were found to increase bond strength of ceramic surfaces to resin cements and composites when compared with control specimens (p-value < 0.01), whereas no significant differences were found in comparison with air-particle abraded surfaces.

High variability can be observed in adhesion values between different analyses, pointing to a need to standardize study protocols and to determine the optimal parameters for each laser type.

## Introduction

Ceramics are the materials of choice for both anterior and posterior dental restorations as they have acceptable longevity and meet patients’ aesthetic requirements [1]. Different compositions are available for dental use, with varying properties that respond to different clinical indications. Zirconia-based ceramics have been reported to provide the best mechanical properties [2,3]. As the translucency of a material is an important parameter for assessing the aesthetic properties of dental ceramics, lithium disilicate and feldspathic ceramics show better aesthetic properties, and so are indicated for anterior crowns and veneers [4,5].

The different systems and protocols for conditioning ceramic surfaces prior to bonding are a topic of interest to clinicians. The adhesion of ceramics to other surfaces involves procedures with varying micromechanical and chemical properties. The bonding of crowns, bridges and veneers to dentin and enamel surfaces must provide sufficient strength for a long-lasting union. Chipping of the ceramic may occur requiring repair; in this context the adhesive properties of repair materials will be important [6]. Lastly, the successful adhesion of orthodontic appliances to ceramic surfaces can also be challenging as prostheses and teeth are subject to constant forces during active treatment [7]. A range of techniques is available for conditioning ceramic surfaces to enhance adhesion. Bond strength can also be influenced by parameters other than surface conditioning such as the application of silane [8], the composition of the ceramic material [9], or the nature and composition of resin cements and composites [10,11].

Airborne particle abrasion with Al_2_O_3_ is a widely used technique for conditioning ceramic surfaces, and is known to provide good bond strength [12,13]. Silica coating is another alternative, which is usually combined with Al_2_O_3_ application [13,14]. An acid can also be applied to the ceramic surface, the most commonly used being hydrofluoric and phosphoric acid [15]. According to the literature, different surface treatments may be recommended for different ceramic materials. In the case of glass ceramics, hydrofluoric acid etching followed by the application of silane coupling agent has been suggested as the gold-standard protocol to create a moistened rough surface for good resin-to-ceramic bonding [16,17]. But in the case of polycrystalline ceramics, which are not silica-based, micromechanical silica-silane bonds cannot be achieved, so some authors recommend air-particle abrasion with Al_2_O_3_ or SiO_2_ of these ceramic surfaces for an enhanced adhesion to resin cements [18,19].

Lasers have been introduced during the last decade as an alternative to traditional methods for ceramic surface treatment. Numerous works have investigated the effects of CO_2_ lasers in continuous or long pulse mode, on shear bond strength of ceramic to other substrates [20,21]. Short pulse lasers such as Nd:YAG, Er:YAG, and Er,Cr:YSGG have also been tested [22–24]. More recently, Ti:Sapphire laser, which provides ultra-short pulses in the femtosecond range, has been introduced, and is considered an optimal alternative as it does not produce any thermal or mechanical damage to the ceramic surfaces [25–28]. However, there is some controversy about the effects of these lasers on the bond strength between ceramic materials and resin cements and composites, with different studies reporting widely differing results [29–32].

The aim of this study was to perform a systematic review and meta-analysis of *in vitro* investigations that have studied the bond strength of laser-conditioned ceramics to resin cements and composites.

## Materials and methods

### Systematic literature search

This systematic review was conducted according to the Preferred Reporting Items for Systematic Reviews and Meta-Analyses (PRISMA) statement [33]. The research question was the following: Do lasers increase the bond strength of composites and resin cements to ceramic materials?

An electronic search for relevant studies was performed in the Pubmed, Embase and Scopus online databases. An electronic search for “grey literature” was also made in the New York Academy of Medicine Grey Literature Report. The reference lists of all the articles identified were also reviewed. The search terms used for all databases were: laser combined with ceramic or porcelain, bond or adhesion and strength, being the search strategy as follows: (laser*) AND (ceramic* OR porcela*) AND (bond* OR adhes*) AND (strength). No publication year or language limit was imposed. The latest search was performed in April 2017. Endnote X7 software (Thompson Reuters, Philadelphia, PA, USA) was used to remove duplicates.

### Study selection

Two experienced researchers (C.B-A and V.G-S) assessed the titles and abstracts of all the articles independently. In the event of any disagreement, a third reviewer (V.P-G) was consulted.

- Inclusion criteria:
  1. Studies that considered bonding to a ceramic substrate.
  2. Studies in which resin cements or composites were used as opposite substrates.
  3. Studies using laser for ceramic surface conditioning (combined or alone) prior to bonding.
  4. Studies including a well designed shear or tensile strength test.
  5. Studies that measured bond strength for at least one laser group.
  6. For quantitative analyses, only studies presenting a baseline control condition or air-particle abrasion surface treatment and laser-irradiated experimental condition were included.
-Exclusion criteria:
  1. *In vivo* or *in situ* studies
  2. Studies testing materials other than resins or composite cements such as brackets, ceramic veneers, dentin, or enamel.
  3. Review studies.
  4. For quantitative analyses, studies using lasers combined with other conditioning methods were excluded.

Papers meeting the elegibility criteria were included in a database and the full texts were analyzed by both reviewers independently.

### Data extraction

Microsoft Office Excel 2013 software (Microsoft Corporation, Redmond, WA, USA) was used to register relevant data drawn from the articles reviewed: publication year, study groups, laser type, laser parameters, sample size, ceramic type, resin cement/composite type, storage conditions, thermocycling and cyclic loading protocols (if any), load applied (mm/min), bond strength test results (MPa) and conclusions (Table in S1 Table).

### Risk of bias and quality assessment

Two reviewers (JM M-C and A.A) assessed the methodological quality of each study independently, using an adapted protocol from an *in vitro* systematic review conducted by Sarkis-Onofre *et al*. [34], based on the articles' description of the following parameters: sample size calculation, adequate control group, laser settings, materials used according to manufacturers’ instructions, surface treatment by single operator, bonding by single operator, and adequate statistical analysis (mean, standard deviation and p-values present).

Each parameter reported by the articles’ authors was marked with a “Y” (yes) for the specific item; if the information was missing, the parameter was marked with an “N” (no). Articles that included only one to three of these items were classified as having a high risk of bias, four or five items as medium risk of bias, and six or seven items as low risk of bias.

### Descriptive statistics and analysis

For quantitative synthesis, the overall mean bond strength (MPa) was calculated for each laser type, for control groups, and for the surface preparation method with air particle abrasion with Al_2_O_3_ (APA). Additional meta-analyses were run, considering each ceramic type independently according to their microstructure (glass, particle-filled glass, and polycristalline). Lastly, quantitative analyses were performed including only polycrystalline ceramics. Studies lacking a control group or APA group, or studies with no standard deviation values, were not included for meta-analysis. Studies in which laser application was combined with other conditioning methods were also excluded. All possible comparisons were made between different laser groups, control groups and/or APA, in the first place, without making distinctions between ceramic types, and in the second place, considering each ceramic type independently. For articles comparing groups treated with the same laser (using different laser settings or different storage protocols), only the group reporting the highest bond strength values was included for meta-analysis.

Inter-group differences between means and their confidence intervals were determined for all the studies included in meta-analysis. A p-value ≤0.05 was considered statistically significant.

Heterogeneity was assessed with Cochran’s Q test, in which a threshold p-value of 0.1 was considered statistically significant, and the I^2^ test, in which values smaller than 50% were considered indicative of low heterogeneity, values between 50–75% moderate heterogeneity, and values greater than 75% indicated high heterogeneity. The DerSimonian-Laird random effects pooling method was used to calculate differences between weighted means [35]. Rosenthal's fail-safe number and funnel plots were used to assess publication bias [36]. Comprehensive Meta-Analysis V.3 (Biostat, Inc) software was used for quantitative synthesis.

The influence of each laser and each ceramic type on the bond strengths of composites and resin cements to ceramic surfaces was analyzed by conducting six different analyses: (1) different lasers versus control groups; (2) different lasers versus APA groups; (3) different ceramics in laser versus control groups; (4) different ceramics in laser versus APA groups; (5) different lasers versus control groups for datasets using polycristalline ceramics; and (6) different lasers versus APA groups for datasets using polycristalline ceramics. Finally, sensitivity analyses were conducted by excluding each study in turn in order to explore the cause of heterogeneity.

## Results

### Search strategy

A total of 635 studies were identified: 185 in Pubmed, 148 in Embase, 302 in Scopus and none in the grey literature database. 210 duplicates were removed and 338 were discarded after reading the title and abstract. The Kappa score for inter-reviewer agreement was 0.87.

The full texts of the remaining 87 articles were read and analyzed. After thorough assessment, 35 articles were excluded for the following reasons: bond strength numerical values not present (2 studies), bonding of materials other than composites or resin cements to the ceramic surfaces (19 studies), or laser used for purposes other than ceramic surface conditioning (14 studies).

**Fig 1:**
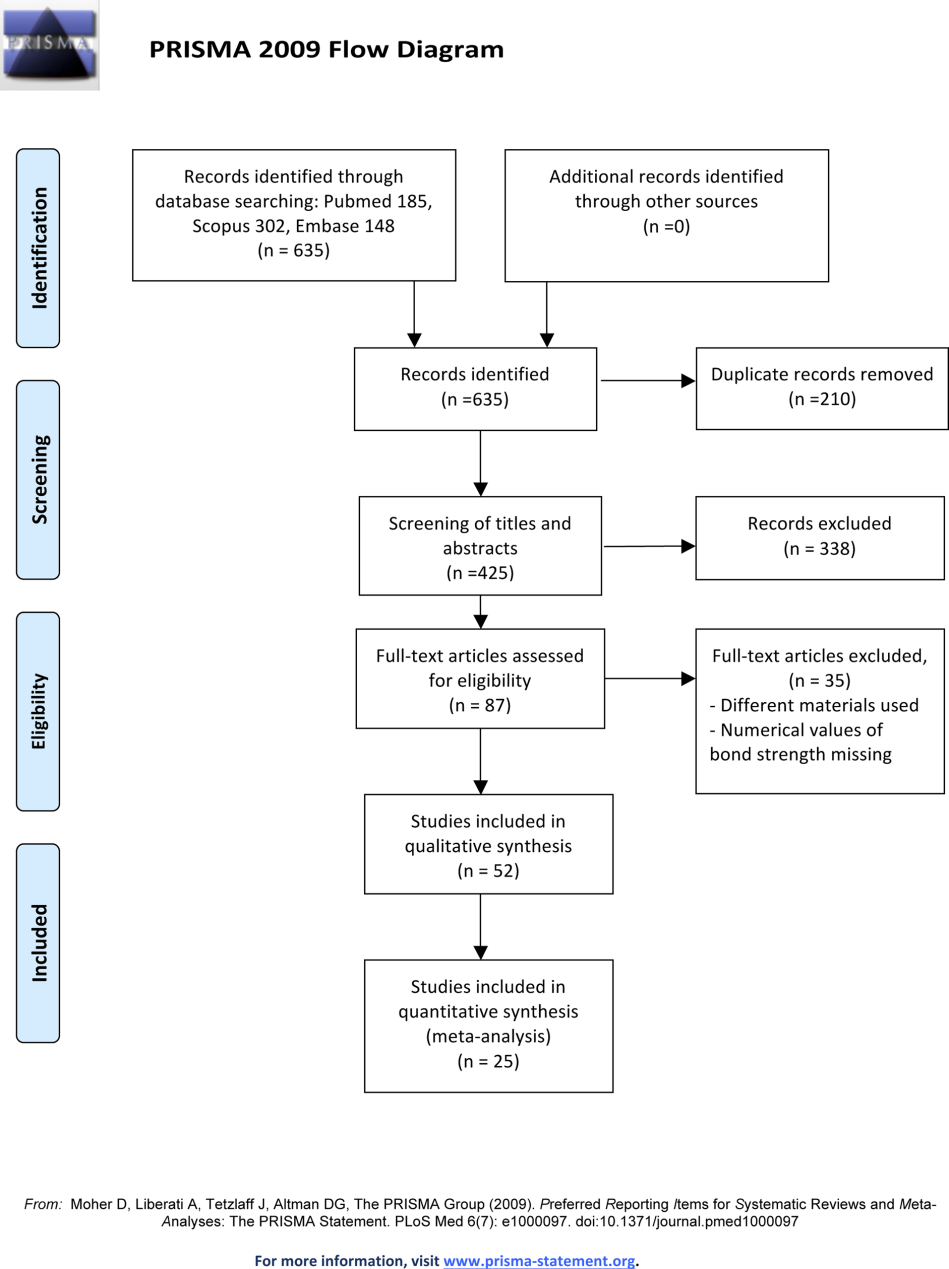
The PRISMA flow diagram. From: Moher D, Liberati A, Tetzlaff J, Altman DG, The PRISMA Group (2009). Preferred Reporting Items for Systematic Reviews and Meta-Analyses: The PRISMA Statement.

Finally, 52 studies met the eligibility criteria and were included for qualitative analysis, whereas 25 articles were included for quantitative synthesis, of which 23 were used for the first meta-analysis, 19 for the second, 23 for the third, 19 for the fourth, 19 for the fifth and 15 for the sixth (Fig 1).

### Qualitative analysis

Six types of lasers were identified in the 52 articles analyzed in this review. A total of 14 studies included CO_2_ laser [21,24,30,32,37–46]; six studies used Er,Cr:YSGG laser [22,30,47–50]; 23 articles included Er:YAG laser groups [9,10,31,32,37,44,51–67]; Femtosecond laser was used in six articles [25,26,53,55,68,69]; Nd:YAG in 18 of the studies [11,24,39,48,52,53,55,56,63,65,66,70–76]; and Yb:YAG laser was used in only one study [29]. The lasers were used with different power outputs, ranging from 400mW to 10W, femtosecond lasers using the lowest power, in contrast with Er:YAG and CO_2,_ which were set at the highest power outputs. Variability was also observed in mean energy settings (4mJ to 500mJ); application time (2 seconds to 2 minutes); and distance, some of the lasers being used in contact mode, whereas other devices were applied at distances ranging from 1 mm to 11 cm. In some studies, the same laser type was used in different groups at different settings. A broad variety of ceramic types was used, zirconia being the most common (36 studies), followed by feldspathic (11 studies), lithium disilicate (5 studies), alumina (3 studies), and leucite glass (1 study). High variability was also found among composites and cements. After the bonding procedure, and prior to bond strength tests, most articles reported storing samples in distilled water for 24 hours at 37°. Many studies carried out thermocycling using different protocols, but only one performed cyclic loading [25]. All the studies performed bond strength tests by means of a shear load at a crosshead speed of 0.5 or 1mm / min, with the exception of five studies, which performed microtensile tests at 0.1 and 0.5 mm / min [29,52,63,75,76].

The S1 Table details the studies selected for analysis, showing the surface treatment groups (including laser types and settings), sample size (n/group), ceramic type, resin cement / composite type or brand, storage conditions, thermocycling and cyclic loading protocols (if any), load applied (mm/min), bond strength test mean values, and standard deviations (if reported) (MPa), and conclusions.

### Meta-analysis

**Fig 2:**
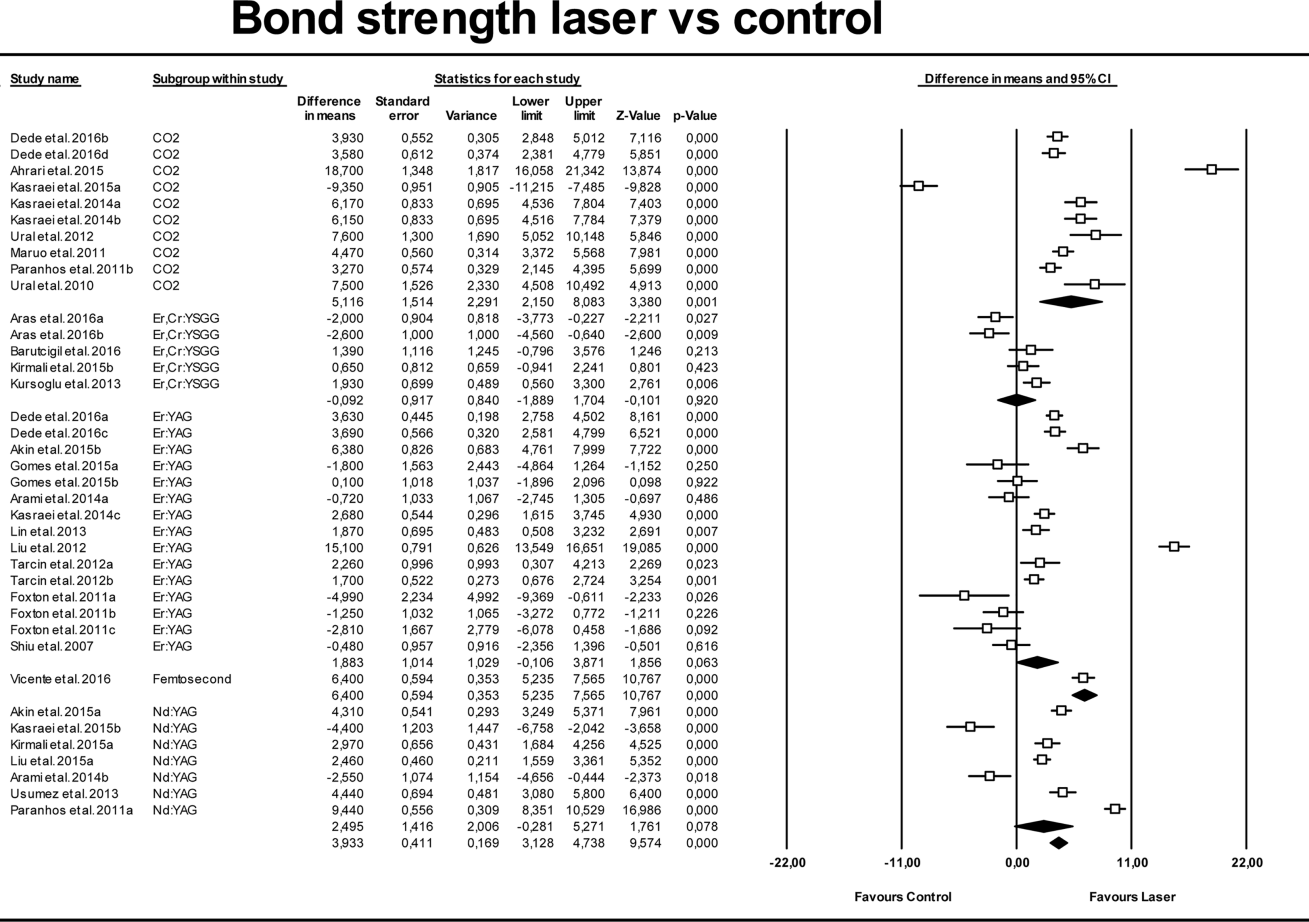
Forest plot summarizing bond strengths obtained by different laser groups versus control groups.

Bond strengths in control groups (no surface treatment) and CO_2_, ErCr:YSGG, Er:YAG, Femtosecond and Nd:YAG lasers were compared; the results are shown in Fig 2. Q and I^2^ tests showed high heterogeneity, the Q-test p-value being Q = 0.000 and I^2^ = 96.4%, so results were analyzed using the random-effects model. Overall values showed that laser treatment increased the bond strength (3.93 MPa [CI 95% from 3.13 to 4.73 Mpa]) of composites and resin cements to porcelain surfaces compared with control groups, with statistically significant difference (p-value <0.01).

When analyzing differences for each individual laser type, a mean difference of 5.1 MPa (95% CI 2.15 to 8.08 MPa) was found between CO_2_ and controls, with statisitical significance (p-value = 0.001). Femtosecond laser showed similar results. For the rest of the lasers, differences in comparison with control groups were not statistically significant, the mean differences being -0.09 MPa for ErCr:YSSG, 1.88 MPa for Er:YAG, and 2.49 MPa for Nd:YAG, with p-values of 0.920, 0.063 and 0.078, respectively. All the models showed high heterogeneity (I^2^>81, Q test p-value = 0.000).

**Fig 3:**
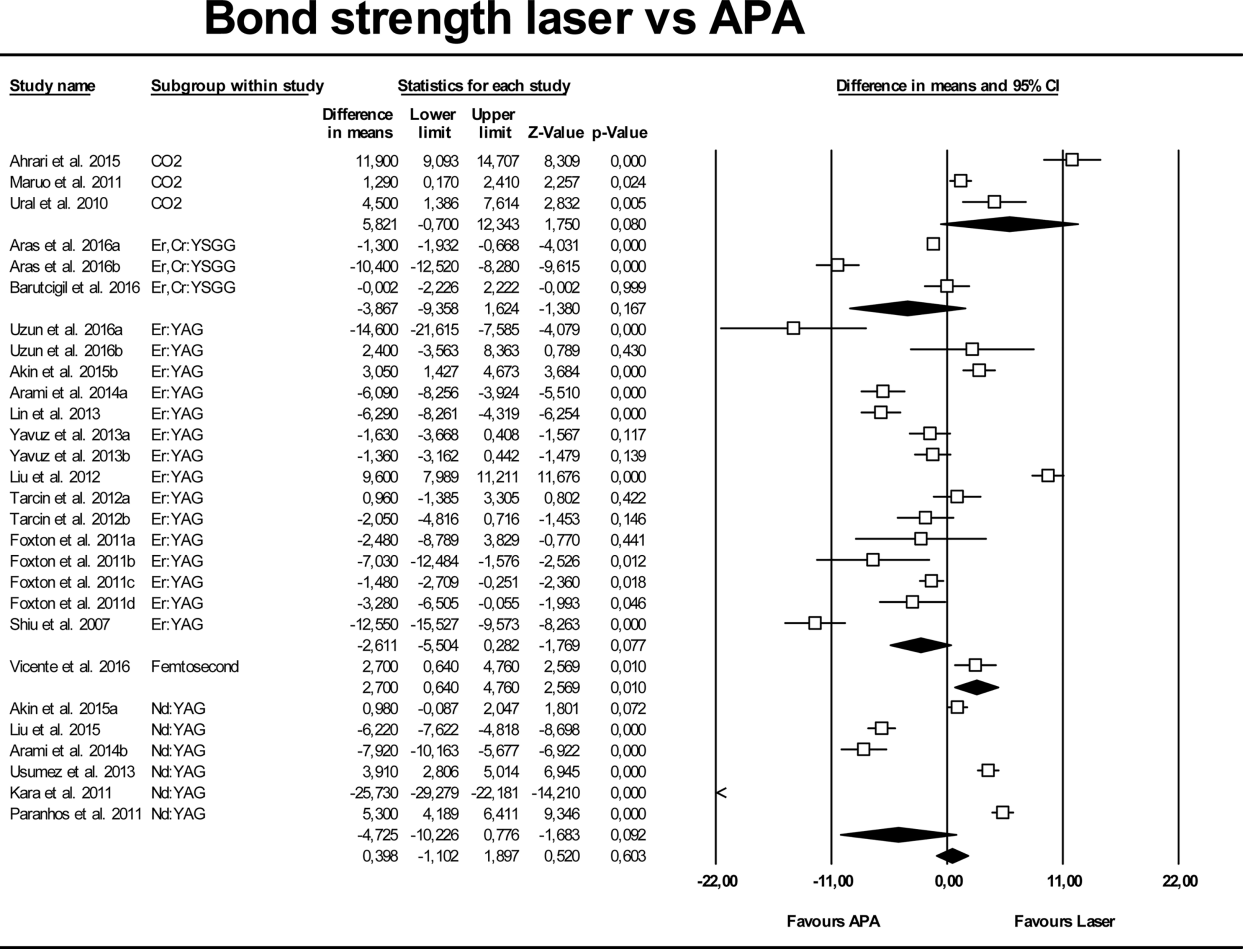
Forest plot summarizing bond strengths of different laser groups versus APA groups (air-particle abrasion).

Fig 3 shows the results of the second meta-analysis, in which laser-conditioning techniques were compared with the APA surface treatment. High heterogeneity was also found for this model (Q test p-value = 0.000; I^2^ = 97.2%), and so the random-effects model was used for analysis. No significant differences were found when the APA mean value was compared with the overall result (p-value = 0.603), the mean difference being 0.39 MPa (95% CI -1.10 to 1.89). No significant differences were found when analyzing each laser type separately.

**Fig 4:**
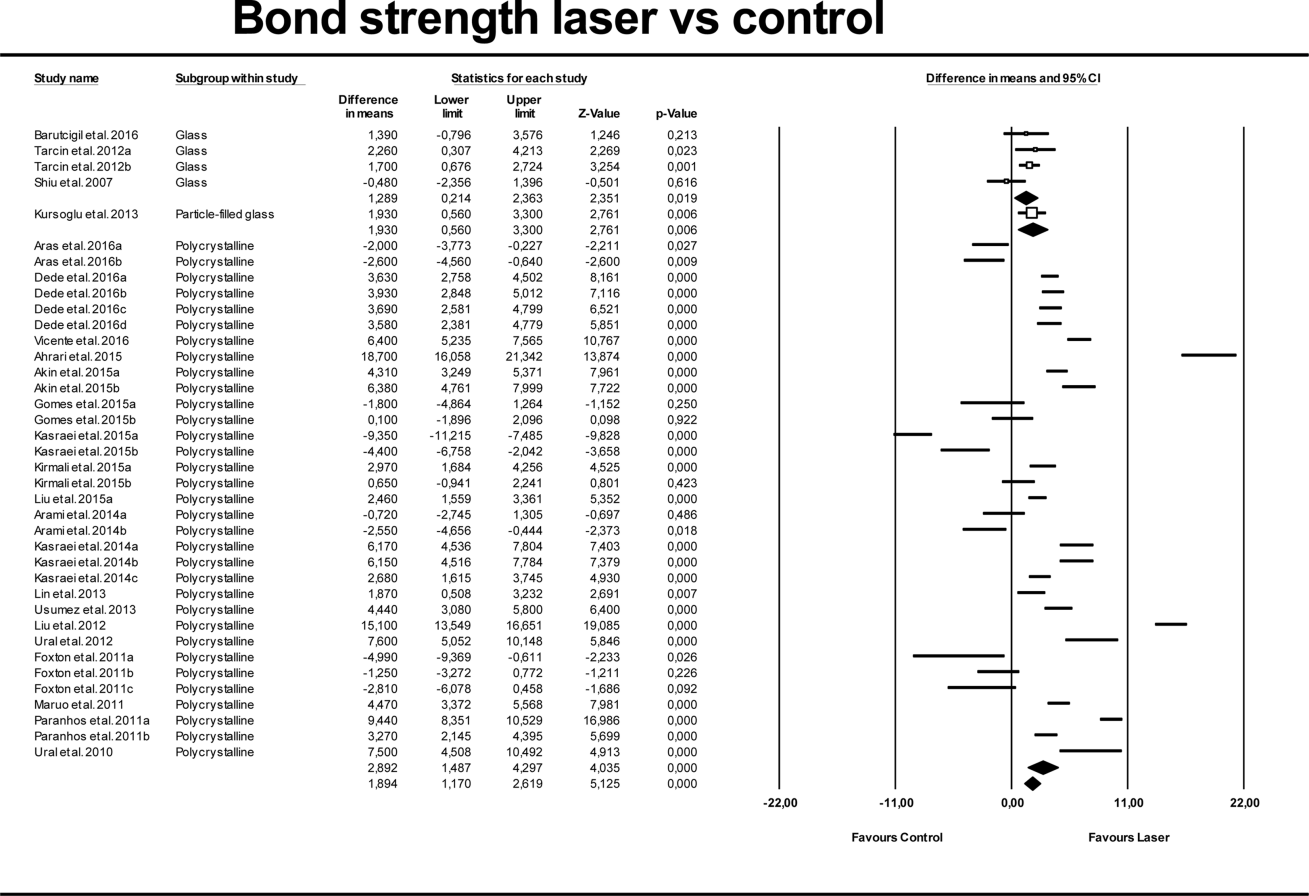
Forest plot summarizing bond strengths obtained by different ceramic types (polycrystalline, particle filled glass, and glass) for laser groups versus control groups.

**Fig 5:**
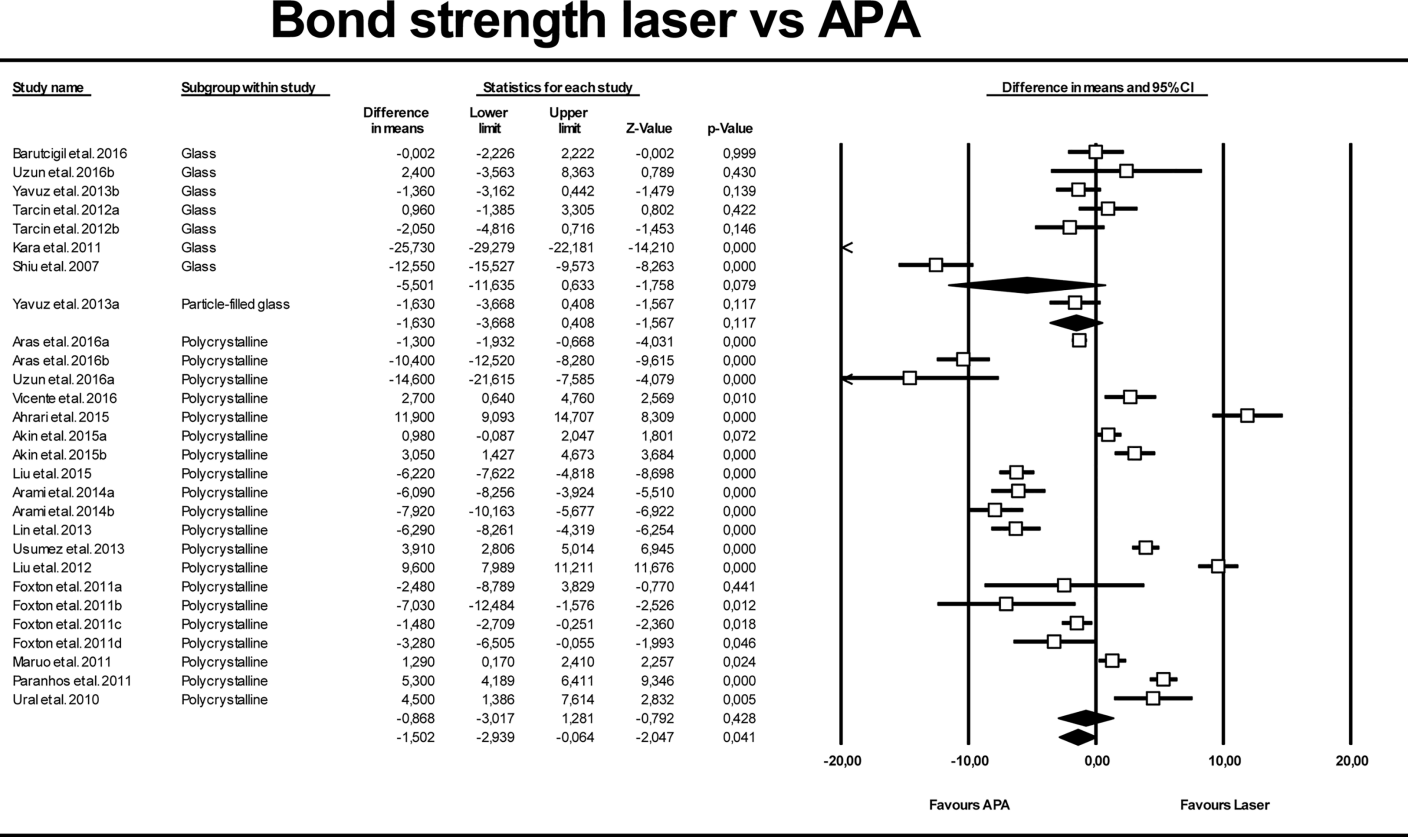
Forest plot summarizing bond strengths obtained by different ceramic types (polycrystalline, particle filled glass, and glass) for laser groups versus APA groups.

Results of the quantitative analysis for lasers versus control and APA groups, considering each ceramic type independently are shown in Figs 4 and 5. These models also showed high heterogeneity (Q = 0.000; I^2^ = 96.38% and 97.26%, respectively). Polycrystalline ceramics showed higher bond strengths than glass and particle-filled glass ceramics, with no significant differences between the three ceramic groups.

**Fig 6:**
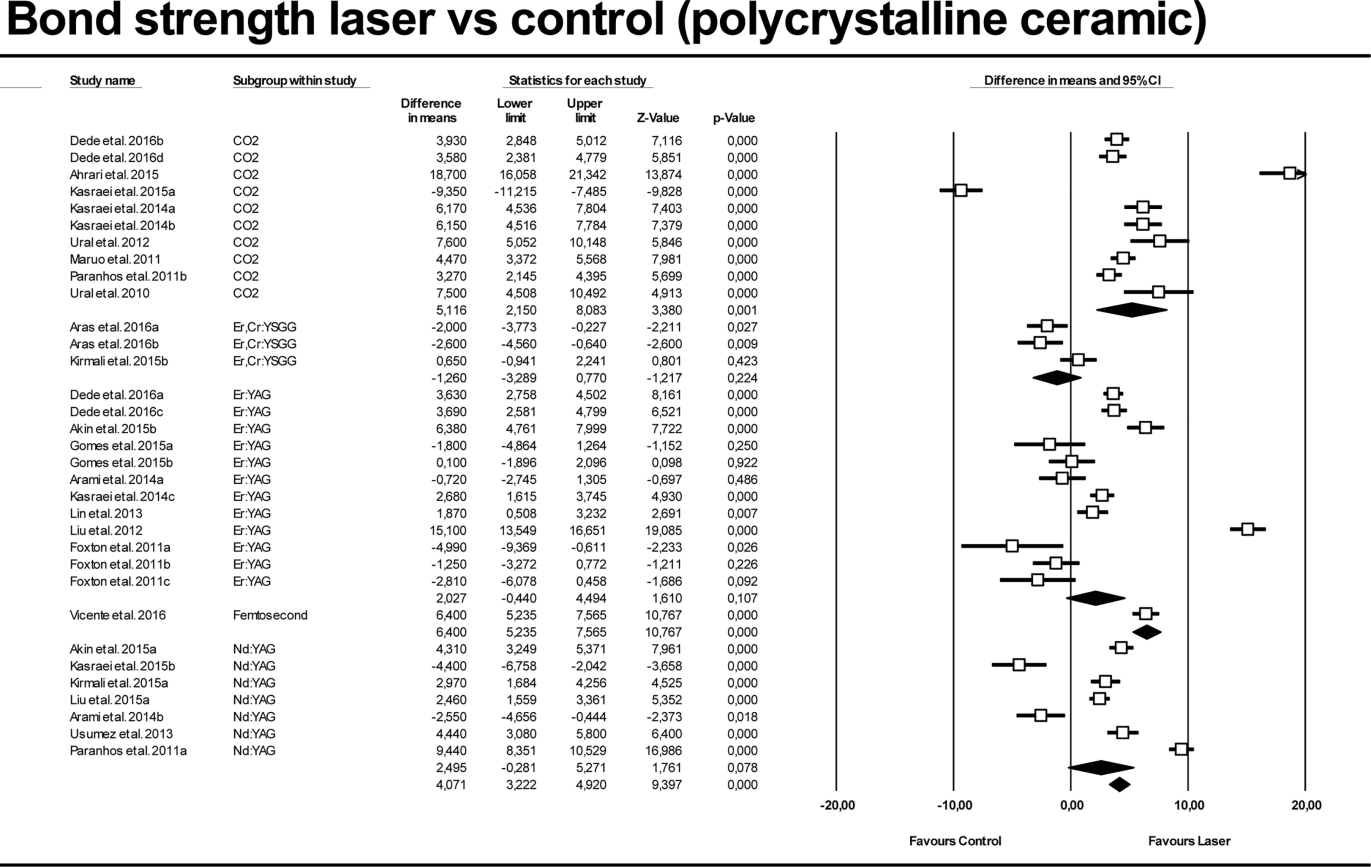
Forest plot summarizing bond strengths obtained by polycrystalline ceramics for laser groups versus control groups.

**Fig 7:**
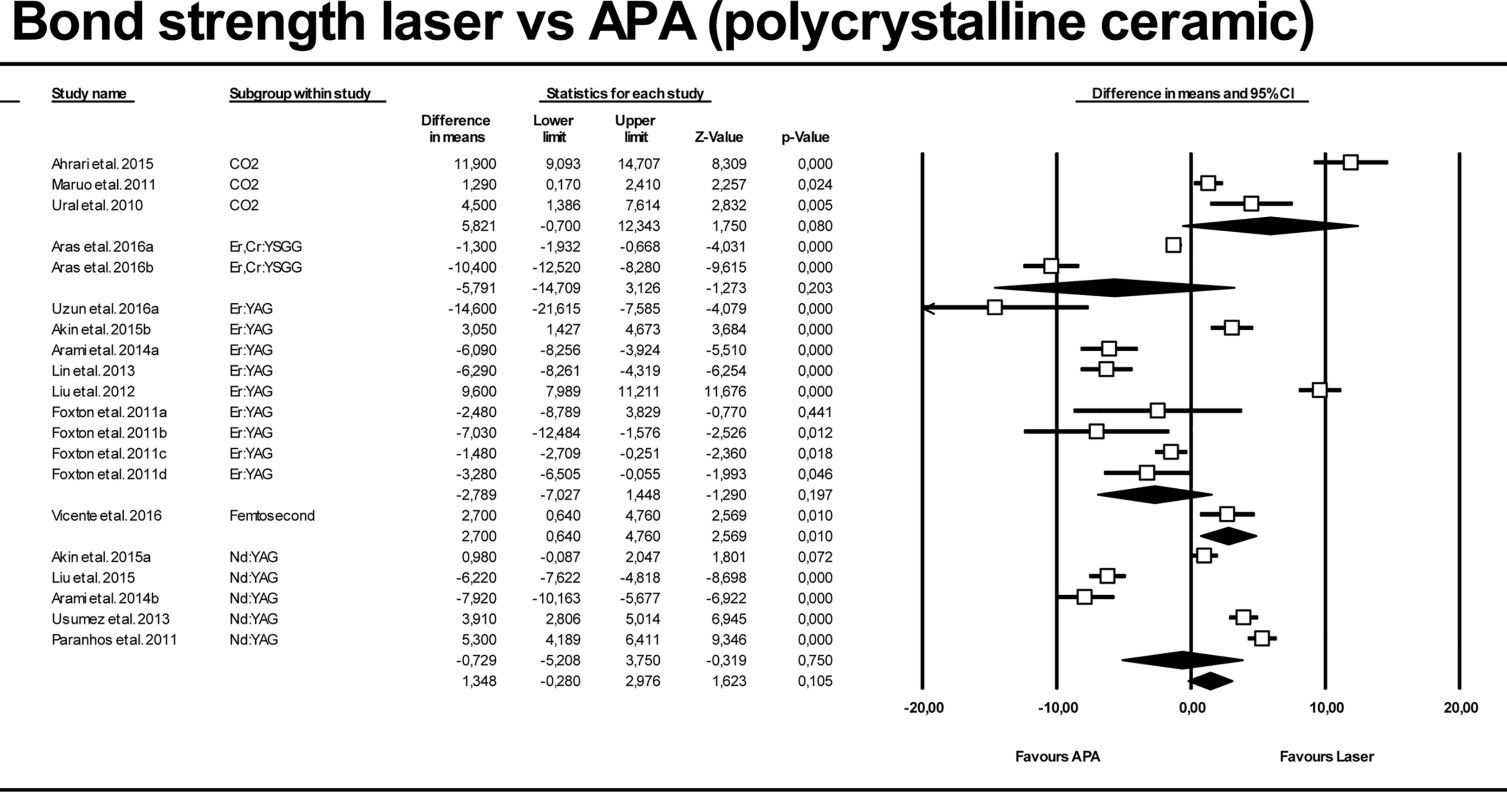
Forest plot summarizing bond strengths obtained by polycrystalline ceramics for laser groups versus APA groups.

Figs 6 and 7 show meta-analysis results for polycrystalline ceramics, lasers versus control and APA groups. High heterogeneity was also found for both models (Q = 0.000; I^2^ = 96.74% and 97.32%). Generally, lasers increased bond strength (4.071 MPa [CI 95% from 3.22 to 4.92 Mpa]) compared with control groups, with statistically significant difference (p-value < 0.01). When evaluating each laser independently, only CO_2_ and femtosecond laser enhanced adhesion significantly (p-value < 0.01). Regarding comparisons between lasers and APA groups when analyzing polycrystalline datasets, only femtosecond laser was found to improve bond strength significantly (2.70 MPa [CI 95% from 0.64 to 4.76 Mpa]; p-value = 0.01). Femtosecond laser estimation was obtained from only one dataset.

Sensitivity analyses showed that none of the studies was found to be the cause for the high heterogeneity (S1 Fig and S2 Fig).

**Fig 8:**
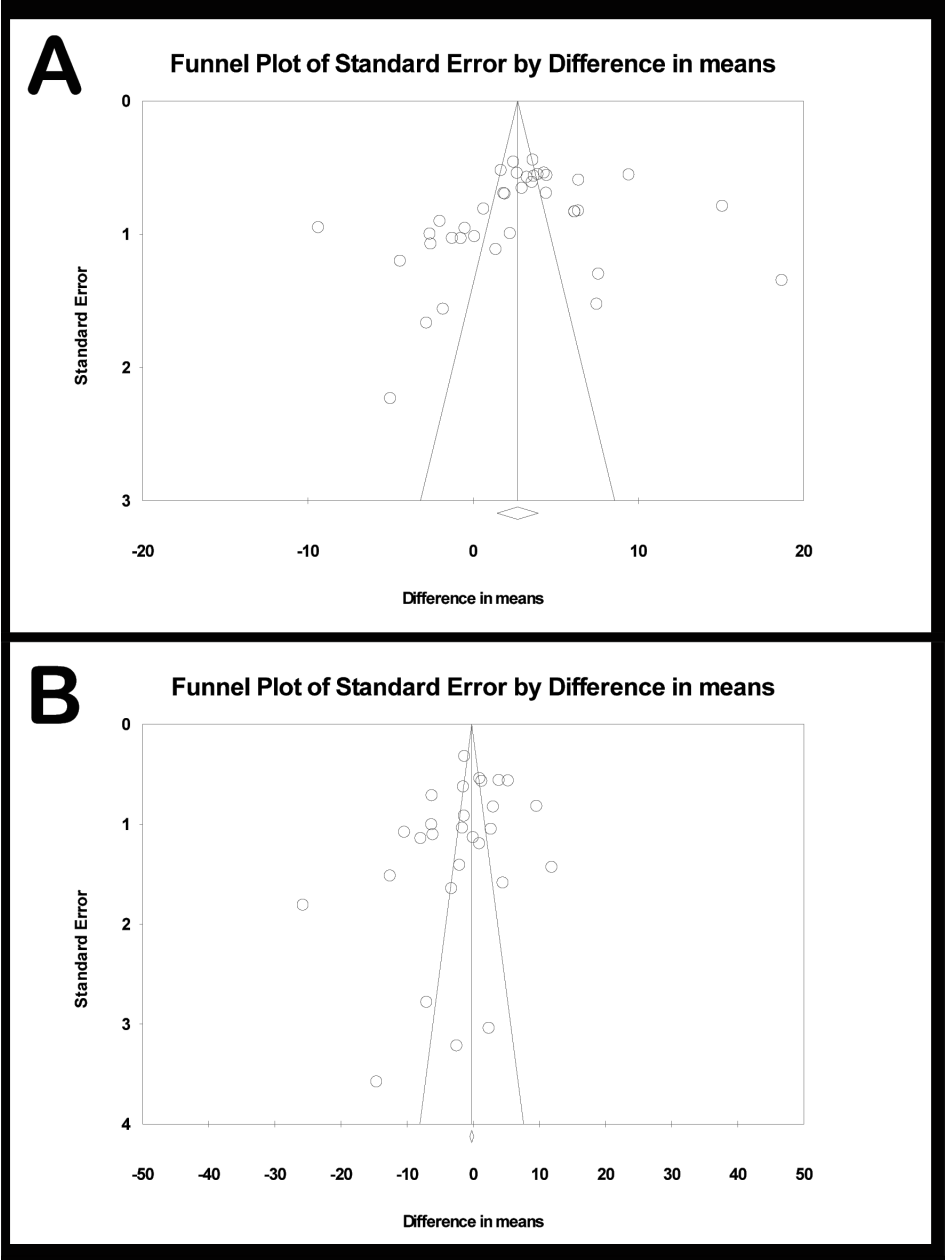
A) Funnel plot of laser versus control meta-analysis. B) Funnel plot of laser versus APA meta-analysis.

Publication bias impact for meta-analyses was found to be low as shown in funnel plots (Fig 8A and 8B), obtaining high (Rosenthal’s) tolerance levels.

### Quality assessment

**Fig 9:**
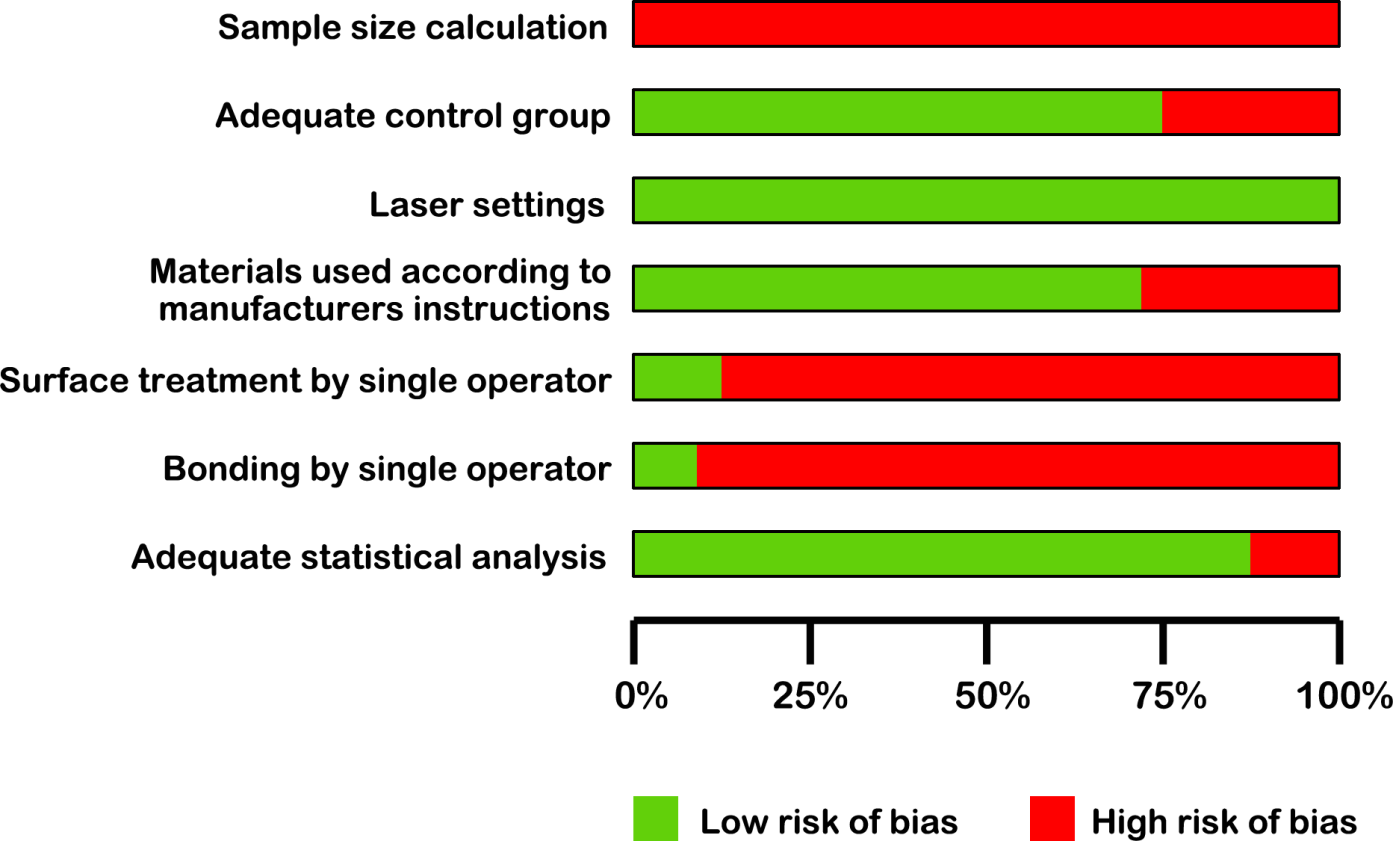
Risk of bias diagram.

According to the parameters applied for quality assessment, 30 studies out of the 52 included in the review presented a medium risk of bias, one of them scored low risk, and the rest of them showed a high risk of bias (Table in S2 Table and Fig 9). On average, positive scores were obtained for the following items: adequate control group, laser settings, materials used according to manufacturers instructions, and adequate statistical analysis.

## Discussion

Surface conditioning with laser devices has been extensively studied in the last few years in an attempt to determine the best laser type and protocol for optimal bonding of adhesive materials to ceramic surfaces. Studies analyzing the performance of lasers on ceramics have taken the form of *in vitro* assays following a wide variety of protocols. In the present systematic review and meta-analysis, 52 publications were thoroughly analyzed, finding high variability. All the studies tested the bond strength (in Mpa) of composites or resin cements to different ceramic surfaces irradiated with laser. Studies testing bond strength of ceramics to other materials such as veneer ceramics, dentin, enamel, metals or orthodontic brackets [23,77,78] were excluded from this review since their behavior on these surfaces is very heterogeneous and has completely different clinical relevance. According to the exclusion criteria, *in-vivo* and *in-situ* studies were not to be included in the review due to the general difficulty of standardization with this kind of study, which could lead to bias. All the publications identified in the search were *in vitro* studies, and so none were excluded for this reason.

Several reviews have been published on the effects of different methods and protocols for ceramic surface conditioning on bond strength [79,80], but none have performed a quantitative analysis of the adhesion results. Inokoshi *et al*. conducted a meta-analysis on bonding to zirconia in which bond strength results in a selection studies were compared in association with different surface treatments, the effects of aging, different cements, and test protocols [81]. But none of the reviews or meta-analyses published to date have focused on laser as a pre-treatment technique.

The studies included in the present review and meta-analysis did not show consensus regarding the efficacy of the different lasers on bond strength to ceramic materials in comparison with conventional methods. Some studies have reported better results for other conditioning techniques compared with lasers, such as silica coating [11,41,43,47,51,57,65,67], air-particle abrasion [30,41,44,49,56,57,59,63,67,71,74], hydrofluoric acid etching [31,50,63,65,67], or grinding with burs [9]. The differences in bond strength found for different surface treatments could be due to the type of ceramic used in each study. Most of the studies that have found that APA increased adhesion investigated bonding to zirconia [30,44,49,56,57,59,71], a finding that concurs with Awliya *et al*. and Blatz *et al*., whose studies recommended air-particle abrasion as the gold standard protocol for polycrystalline ceramics [18,19]. Studies that obtained higher adhesion values for the groups treated with hydrofluoric acid used feldspathic ceramics [27,46,59,61,63], these results supporting claims that this treatment is the gold standard for glass ceramics [16,17].

But other investigations have found lasers to increase the bond strength of composites and cements to ceramics compared with other methods [21,25,26,29,38,39,43,46,48,53,61,70,75,76]. Nevertheless, these studies compared different lasers [24,30,32,37,39,44,48,52,53,55,56,59,65,66], with results that express a lack of consensus as to which laser type is more effective. This variability in results might be due to the disparity among studies in terms of the laser device settings used, the composition of the ceramic materials, composites or cements, storage conditions, and thermocycling/cyclic loading protocols. Furthermore, the laser groups in some of these studies applied laser alone to the ceramic surface, whereas others combined laser irradiation with other techniques in multiple combinations, variations that could also affect bond strength outcomes.

So, altering laser parameters could affect the performance of these devices when conditioning surfaces. Some of the studies analyzed in the present review compared different parameters for a single laser, modifying power outputs, energy, repetition rates, pulse duration or application time, with varying results. Some authors found that changing laser settings significantly affected bond strength [30,31,38,42,50,54], while other studies reported no significant differences [29,37,43,49,59,71,72].

The type of ceramic material is also an issue to take into consideration when evaluating bond strength results, since different ceramics can exhibit different mechanical behaviors depending on their composition. Most of the studies reviewed here only tested one ceramic type, zirconia being the most commonly used. Few studies have compared different ceramic materials. El Gamal *et al*. and Yavuz *et al*. compared lithium disilicate with zirconia ceramic, obtaining higher bond strength results for zirconia in the laser groups [21,55]. Uzun *et al*. found higher values for feldspahic ceramic in the laser group compared with zirconia [9]. Yavuz *et al*. and Foxton *et al*. also compared two different ceramics (lithium disilicate versus feldspathic, and aluminum oxide versus zirconia respectively), but did not obtain significant differences between the materials [61,64]. In the present study, meta-analyses were performed considering each ceramic type independently (polycrystalline, glass and particle-filled glass ceramics). Although polycrystalline ceramics were found to obtain higher bond strength values than the other ceramic types, there were no significant differences among them. On the basis of these results, the ceramic type variable can be dismissed as the cause for the high heterogeneity found in the meta-analyses.

The studies also showed high variability in the composites and cements assayed, which could potentially affect bond strength. Several studies comparing different cements have concluded that phosphate monomer-containing resin cements produce higher bond strengths [11,57,58,64].

Other parameters that could alter bond strength are the different storage conditions and cycling procedures used in different studies. Most of the studies stored samples in distilled water at 37° for 24 hours prior to bond strength tests. However, some studies changed this protocol to 48 or 72 hours, or even several weeks or months. Storage condition protocols could affect the bond between surfaces and some studies have analyzed the effects of increasing storage time, since this variable is believed to simulate the aging of the bonded interface. Tanis *et al*., Esteves-Oliveira *et al*., and Aras *et al*. found no differences in adhesion results in laser groups when samples were stored for 24 hours versus one month, six months and 14 days respectively [11,29,47]. However, Foxton *et al*. did find significant differences when samples were stored for 24 hours versus 6 months [64]. Some studies have demonstrated that the durability of the bonded interface depends on the cement and primer composition. In this sense, MDP monomer-containing bonding agents have been proved to be resistant to water degradation [82], whereas cements with other compositions are affected by aging as a result of exposure to water [83]. Surface treatment can also affect the resistance to degradation, as stated in the meta-analysis conducted by Aurélio *et al*. in which the APA technique was found to increase the long-term resistance to aging of zirconia ceramics [84].

Lastly, some differences in bond strength could be due to different thermocycling or cyclic loading protocols applied in studies evaluating bond stability. Vicente *et al*. concluded that cyclic loading significantly decreased bond strength when treating zirconia surfaces with femtosecond laser [25]. Mechanical degradation testing was not performed in any of the other studies included in the present review. This fact may be a source of bias, as this variable can significantly affect bond strength. When conducting bond strength assays, it is important to simulate the real clinical conditions to which dental restorations are subject. Thermocycling was performed in some of the studies reviewed, using different protocols with varying numbers of cycles, temperatures, and duration. Only four of these studies analyzed thermocycling effects on bond strength outcomes when treating ceramic with laser devices, two of them finding that the procedure decreased adhesion efficacy [10,45], while the other two did not find significant differences [59,71].

This systematic review did not consider different types of bond failure, since not all the studies reviewed assessed this variable, and when they did, different indices were applied. Moreover, this is a factor with a certain element of subjectivity as it depends on the criteria applied by the individual operator.

Differences in terms of laser types and protocols have been an object of research for many years. The first publication included in this review dates from 2005, and used Nd:YAG laser on aluminum oxide ceramic [75]. Nd:YAG, Er:YAG and CO_2_ lasers have been widely studied from the first publications up to the present, with a noticeable increase in the number of articles per year.

Femtosecond lasers have been the latest addition, and have investigated in the last few years, reporting good results in terms of bond strength. Some authors have tested femtosecond lasers comparing them with conventional techniques or with other lasers, concluding that femtosecond laser increases the bond strength of resin cements and composites to ceramic surfaces [26,55]. Other studies have focused on determining the best parameters and protocols for obtaining optimal results with this laser. Vicente *et al*. compared different irradiation patterns [25]; Akpinar *et al*. used the laser beam at different angulations [68]; and Apkinar *et al*. created different shapes on the ceramic surfaces [69]. Different results were obtained by these authors for each protocol, which confirms that bond strength greatly depends on factors other than the laser type used.

The systematic review’s quantitative analyses included 25 studies. The first meta-analysis only included studies with a control group in order to obtain absolute results regarding laser performance. In the second meta-analysis, laser groups were compared with those treated with air-particle abrasion since this conditioning technique is widely used by clinicians and was the most commonly tested in the studies reviewed. In order to avoid bias, datasets in which lasers were combined with other methods were excluded. Furthermore, for studies using different protocols for the same laser, only the datasets of laser groups reporting the highest bond strengths were used. Although this method could lead to overestimation in the meta-analysis results, protocols used in datasets with lower bond strength values were assumed to be dismissed because of their failure to achieve good adhesion.

These meta-analyses showed high heterogeneity. Sensitivity analyses were conducted in order to determine if any particular studies were responsible for generating this heterogeneity. But none of the studies were found to be a cause of heterogeneity.

Two meta-analyses considered each ceramic type independently (polycrystalline, glass and particle-filled glass ceramics). Although polycrystalline ceramics were found to obtain higher bond strength values than the other ceramic types, no significant differences were found among them. These results dismiss the ceramic type variable as a source of the high heterogeneity found in the meta-analyses.

In light of the results of sensitivity analyses and of the meta-analyses comparing different ceramic types, heterogeneity can be attributed to other variables such as the different laser settings used in the studies, different cements, or varying methodologies.

Risk of bias was found to be high in 21 of the studies included in the present review and medium in 30, which, together with high heterogeneity, highlights the need for standardized methods. The risk of bias identified can be attributed mainly to the lack of information regarding the number of operators performing the experiments and sample size calculations. Sample sizes were similar in all the studies although no power analyses were performed. Single versus multiple operators could affect the results of the studies, especially at the bonding stage. Another cause of medium or high risk of bias, which could also affect the bond strength results, was that it was not stated whether or not the bonding agents had been used according to the manufacturer’s instructions, which may have led to some failure to obtain optimal efficacy.

Overall, According to the present results, some lasers can improve the bond strength of composites and cements to ceramic surfaces. Although femtosecond lasers have not widely been studied, the bond strngth results obtained by these devices were positive compared with other lasers. Focusing on the lasers available at present, according to the present meta-analysis, CO_2_ laser irradiation applied for 10 seconds to ceramic surfaces when bonding composites or resin cements would appear to produce positive results.

The variability of results found in the present review points to the need for further studies, and for the standardization of protocols. Standardized protocols should attempt to reproduce clinical conditions, in order to determine each specific laser’s parameters for producing optimal bond strength. To do this, the choice of bonding agent should be optimized; some studies have shown that phosphate monomer-containing cements are more effective and resist aging better. In addition, a combination of shear and tensile mechanical tests should be performed to determine the bond strength between interfaces, as bonds are clinically subject to a combination both forces. Lastly, the application of degradation protocols should be included in all studies in order to simulate the chemical effects of saliva, and the temperature variations and masticatory forces that restorations may be subject to.

## Conclusions

Although the studies included in the present meta-analyses showed high heterogeneity, lasers may be recommended as an alternative technique for treating ceramic surfaces prior to bonding composites or resin cements.

The present results show that lasers provide higher bond strengths compared to non-treated surfaces but there are no significant differences in comparison with air-particle abrasion methods.

It is necessary to standardize study protocols and determine the adequate parameters of each laser type in order to determine which is the most efficient surface conditioning technique for producing adequate bond strengths.

## Supporting information

S1 Table Articles included in the systematic review and detailed information about methodology, results and conclusions of each publication. (DOCX)

S2 Table Risk of bias assessment. (DOCX)

S1 Fig Forest plot summarizing the results of sensitivity analysis for laser versus control groups. (TIF)

S2 Fig Forest plot summarizing the results of sensitivity analysis for laser versus APA groups. (TIF)
