# Supplementary material for: The effects of lasers on bond strength to ceramic materials: A systematic review and meta-analysis
Source: PLoS One. 2018 Jan 2;13(1):e0190736. doi: 10.1371/journal.pone.0190736 (PMC5749860; doi:10.1371/journal.pone.0190736)
Supplement: S1 Table — (DOCX) [file pone.0190736.s001.docx]

Articles included in the systematic review and detailed information about the surface treatment applied to the ceramic, sample size (n/group), ceramic type, bonding material used, storage conditions, shear/tear testing machine crosshead speed, study results (bond strength in MPa –mean and standard deviation-) and conclusions

| **PAPER** | **SURFACE TREATMENT** | **n/**  **group** | **CERAMIC** | **RESIN CEMENT / COMPOSITE** | **POST-BONDING STORAGE / THERMOCYCLING / CYCLIC LOADING** | **Crosshead speed**  **mm/min** | **BOND STRENGTH RESULTS (MPa)**  **Mean / Standard Deviation**  ***Groups with different letters are statistically different.** | **CONCLUSIONS** |
| --- | --- | --- | --- | --- | --- | --- | --- | --- |
| El Gamal et al.  2017  **[21]** | - Control (C)  - CO_2_ 5W + 9% HF acid + Silane (CO_2_+HF+S)  - HF+S  - CO_2_+APA+S  - APA + S | 5 | - lithium disilicate (L)  - zirconia (Z) | Composite resin (Filtek) | Distilled water 37º/24h | 1 | - L+C **7.6 (1.92)**  - Z+C **11.5 (3.02)**  - L+CO_2_+HF+S **16.71 (4.04)**  - L+HF+S **16.90 (6.42)**  - Z+CO_2_+APA+S **30.16 (4.95)**  - Z+APA+S **26.20 (2.89)** | CO_2_ increases bond strength of composite to zirconia |
| Zanatta et al.  2017  **[70]** | - Control (C)  - APA Al_2_O_3_ (APA)  - APA + silica coating (SC)  - Nd:YAG 1mm/100mJ/60s | 13 | zirconia | Resin cement  - Rely X U200 (R)  - Bifix SE (B) | Distilled water 1 week | 1 | - C+R **7.28 (2.75)^Aa^**  - APA+R **13.31 (3.92)^Ba^**  - SC+R **18.05 (3.89)^Ca^**  - Nd:YAG+R **20.99 (8.17)^Ca^**  - C+B **6.82 (2.77)^Aa^**  - APA+B **12.03 (2.86)^ABa^**  - SC+B **17.32 (4.76)^Ba^**  - Nd:YAG+B **22.16 (6.20)^Ca^** | Nd:YAG laser improved the bonding performance of both resin cements tested, being  similar to silica coating for RelyX U200.  RelyX U200 performed similarly to Bifix SE |
| Ahrari et al.  2016  **[38]** | - Control  - APA Al_2_O_3_  - Universal Primer  - CO_2_ 30mm/10W/10mJ/10sec  - CO_2_ 30mm/10W/14mJ/10sec  - CO_2_ 30mm/20W/10mJ/10sec | 15 | zirconia | Resin cement (Clearfil) | Distilled water/24h/  Room temp | 1 | -Control **9.4 (1.8)^a^**  -APA **16.2 (2.6)^b^**  -Primer **22.8 (5.7)^c^**  - CO_2_ 10W/10mJ **18.1 (3.1)^b^**  - CO_2_ 10W/14mJ **27.4 (3.7)^d^**  - CO_2_ 20W/10mJ **28.1 (4.9)^d^** | CO_2_ can improve bond strength of resin cement to zirconia |
| Aras et al.  2016  **[47]** | - Control (C)  - Ceramic Primer (CP)  - Silica Coating (SC)  - Silica Coating + Silane (SCS)  - APA Al_2_O_3_ (APA)  - APA + CP (APACP)  - Er,Cr:YSGG (ER)  - Er,Cr:YSGG + CP (ERC)  *Er,Cr:YSGG 1mm/3W/30s | 5 | zirconia | Resin cement  - Panavia F (P)  - RelyX ARC (R) | Distilled water 37º  - 24h  - 6 months | 1 | - C+R+24h **3.8 (2.2)^Bd^**  - CP+R+24h **11.3 (7.8)^Bbc^**  - SC+R+24h **12.9 (1.4)^Bb^**  - SCS+R+24h **21.1 (8.6)^Ba^**  - APA+R+24h **2.5 (0.6)^Bcd^**  - APACP+R+24h **12.0 (5.5)^Bb^**  - ER+R+24h **1.2 (0.4)^Bd^**  - ERC+R+24h **13.0 (5.8)^Bb^**  - C+R+6m **1.5 (1.3)^Ad^**  - CP+R+6m **10.4 (4.8)^Abc^**  - SC+R+6m **17.0 (7.3)^Ab^**  - SCS+R+6m **28.7 (6.4)^Aa^**  - APA+R+6m **6.5 (3.6)^Acd^**  - APACP+R+6m **18.9 (5.5)^Ab^**  - ER+R+6m **1.6 (0.4)^Ad^**  - ERC+R+6m **17.5 (4.4)^Ab^**  - C+P+24h **2.5 (2.0)^Bbc^**  - CP+P+24h **3.6 (1.9)^Bbc^**  - SC+P+24h **3.2 (0.9)^Abc^**  - SCS+P+24h **9.9 (5.0)^Aa^**  - APA+P+24h **10.9 (2.4)^Ba^**  - APACP+P+24h **9.9 (3.1)^Aa^**  - ER+P+24h **0.5 (0.3)^Ac^**  - ERC+P+24h **6.6 (5.9)^Aabc^**  - C+P+6m **7.1 (1.4)^Abcd^**  - CP+P+6m **10.6 (3.1)^Abc^**  - SC+P+6m **5.0 (1.1)^Acde^**  - SCS+P+6m **10.9 (4.3)^Abc^**  - APA+P+6m **18.7 (2.9)^Aa^**  - APACP+P+6m **12.9 (2.8)^Aab^**  - ER+P+6m **0.5 (0.1)^Ae^**  - ERC+P+6m **3.2 (2.2)^Ade^** | Laser treatment was not sufficient to increase SBS between zirconia and resin cements. Tribochemical silica coating followed by primer achieved the highest SBS values. |
| Barutcigil et al.  2016  **[22]** | - Control (C)  - Silica Coating (SC)  - APA Al_2_O_3_ (APA)  - 10% HF acid (HF)  - Universal adhesive (UA)  - Er,Cr:YSGG 1mm/2W/200mJ/20s | 10 | feldspathic | Resin cement (Rely X U200) | Distilled water 37º/24h | 0.5 | - C **7.751 (2.336)^a^**  - SC **8.757 (3.148)^ab^**  - APA **9.139 (2.428)^ab^**  - HF **10.144 (2.510)^ab^**  - UA **10.728 (3449)^b^**  - Er,Cr:YSGG **9.137 (2.642)^ab^** | Surface treatments of ceramic could improve bond strength to resin cements. No significant differences were found between the surface treatment groups |
| Dede et al.  2016  **[37]** | -LS Control  -LS ErYAG 10mm/6W/300mJ/5s  -LS CO_2_ 2W/20mJ/10sec  -LS CO_2_ 4W/20mJ/10sec  -SS Control  -SS ErYAG 10mm/6W/300mJ/5s  -SS CO_2_ 2W/20mJ/10sec  -SS CO_2_ 4W/20mJ/10sec | 10 | Zirconia  - (LS) Long sinter  - (SS) Short sinter | Composite resin discs-Resin cement  (Panavia 2.0) | Distilled water 37º/24h | 1 | -LS Control **13.54 (0.70)^aa^**  -LS ErYAG **17.17 (1.22)^ab^**  -LS CO_2_ 2W**17.47 (1.60)^ab^**  -LSCO_2_ 4W **16.57 (1.31)^ab^**  -SS Control **12.33 (0.96)^ba^**  -SS ErYAG **16.02 (1.51)^ab^**  -SS CO_2_ 2W **15.49 (1.28)^bb^**  -SS CO_2_ 4W **15.91 (1.68)^ab^** | Variations in sintering time may influence the SBS of zirconia. The CO2 and Er:YAG laser irradiation techniques achieved sufficient SBS between zirconia and resin cement. |
| Esteves-Oliveira et al.  2016  **[29]** | - APA Al_2_O_3_ (APA)  - Silica coating (SC)  - Yb:YAG 9W/L10  - Yb:YAG 9W/L20 | 63/72/71/63/44/49/52/59 | zirconia | Silane + composite resin | Distilled water 37º  - 24h  - 1 month | 0.5 | - APA 24h **22^A^**  - SC 24h **20.8^A^**  - Yb:YAG/L10 24h **42.3^B^**  - Yb:YAG/L20 24h **37.9^B^**  - APA 1m **20.1^A^**  - SC 1m **19.0^A^**  - Yb:YAG/L10 1m **42.3^B^**  - Yb:YAG/L20 1m **34.3^B^** | Laser irradiation significantly increases bond strength of zirconia ceramic |
| Uzun et al. 2016  **[9]** | - Grinding with burs (C)  - APA Al_2_O_3_ (APA)  - Er:YAG 2W/200mJ/10s | 10 | - Zirconia (Z)  - Feldspathic (F) | silane + resin composite (Grandio SO) | Distilled water 37º/24h | 1 | - Z+C **68.4 (10.4)^aA^**  - Z+APA **31.0 (10.0) ^aB^**  - Z+Er:YAG **16.4 (5.3)^aC^**  - F+C **42.3 (11.3)^bA^**  - F+APA **25.2 (5.9)^aB^**  - F+ Er:YAG **27.6 (5.6)^bB^** | Er:YAG laser irradiation was the least effective technique to rough the zirconia surface, but it may be an alternative for conditioning feldspathic ceramic. |
| Vicente et al.  2016  **[25]** | - Control (C)  - APA Al_2_O_3_  - Silica coating (SC)  - FS laser step 20μ (FS20)  - FS laser step 40μ (FS40) | 15 | zirconia | Resin cement (Clearfil) | - Water storage (W)  - Cyclic loading 90N (50000 cycles, 3 cycles/s) (CL) | 0.5 | - C+W **4.4 (1.3)^a^**  - C+CL **3.1 (0.5)^a^**  - APA+W **8.1 (3.6)^b^**  - APA+CL **7.2 (3.4)^b^**  - SC+W **9.5 (2.3)^bc^**  - SC+CL **7.9 (1.7)^b^**  - FS20+W **10.8 (1.9)^c^**  - FS20+CL **8.5 (1.0)^b^**  - FS40+W **10.7 (1.4)^c^**  - FS40+CL **7.7 (0.4)^b^** | Cyclic loading decreases the adhesive effectiveness of the  zirconia-resin interface when any of the surface treatments are applied, except APA, which is not affected by cyclic loading.  Femtosecond laser irradiation and tribochemical silica coating improve the bond strength of  the zirconia-resin interface. |
| Vicente Prieto et al. 2016  **[22]** | - Control (C)  - APA Al_2_O_3_ (APA)  - Silica Coating + Silane (SC)  - FS Laser 100mm/4mJ | 15 | zirconia | Resin cement (Clearfil) | Distilled water 37º/72h | 0.5 | - C **4.4 (1.3)^a^**  - APA **8.1 (3.6)^b^**  - SC **9.5 (2.3)^bc^**  - FS **10.8 (1.9)^c^** | FS laser SC improve the adhesive effectiveness of the zirconia-resin cement interface, with early SBS in the FS group |
| Yenisey et al. 2016  **[51]** | - Control (C)  - Silane (S)  - Silano-Pen (SP)  - APA Al_2_O_3_ (APA)  - APA+Silane (APAS)  - APA+Cojet Silica coating+Silane (APACo)  - APA+Rocatec silica coating+silane (APARo)  - APA+AP diamond+silane (APAD)  - APA + Silano-Pen (APASP)  - APA+Er:YAG+silane (Er:YAG)  *Er:YAG 6W/120mJ/5s | 10 | Zirconia:  - (LS) Long sintered  - (SS) Short sintered | Composite resin (Filtek) + Panavia F | Distilled water 37º/24h  Thermocycled for 6000 cycles between 5-55º/30sec | 1 | - LS+C **4.62 (0.62)^Aa^**  - LS+S **5.55 (0.74)^Aabc^**  - LS+SP **6.51 (0.94)^Abc^**  - LS+APA **4.89 (1.32)^Aab^**  - LS+APAS **7.26 (1.33)^Ac^**  - LS+APACo **11.19 (1.44)^Ad^**  - LS+APARo **4.89 (0.65)^Aab^**  - LS+APAD **5.70 (0.96)^Aabc^**  - LS+APASP **6.10 (0.52)^Abc^**  - LS+ Er:YAG **6.70 (0.66)^abc^**  - SS+C **4.70 (0.63)^Aa^**  - SS+S **5.59 (0.63)^Aab^**  - SS+SP **5.71 (0.75)^Aab^**  - SS+APA **5.91 (0.73)^Aab^**  - SS+APAS **7.36 (1.26)^Abc^**  - SS+APACo **13.36 (2.41)^Ad^**  - SS+APARo **6.01 (1.62)^Aab^**  - SS+APAD **6.54 (1.36)^Abc^**  - SS+APASP **9.22(1.47)^Bcd^**  - SS+ Er:YAG **6.70 (1.03)^Abc^** | Variation in sintering time influenced the shear bond strength of zirconia ceramics also surface treatment techni- ques did.  The 30 mm tribochemical silica coating (Cojet)/MDP silane agent coupling (APACo) system significantly increased the shear bond strength of resin cement to zirconia ceramics |
| Akhavan Zanjani et al.  2015  **[30]** | - APA Al_2_O_3_  - CO_2_ 4W/50sec  - Er,Cr:YSGG 1mm/3W/50sec  - Er,Cr:YSGG 1mm/2W/50sec | 14 | zirconia | Resin cement (Panavia F2.0) | Incubated 48h/37º/  98% humidity | 0.5 | -APA **37.3066 (5.57471)^a^**  - CO_2_ **29.0802 (6.59420)^b^**  - Er,Cr 3W **27.5204 (4.85286)**^b^  - Er,Cr 2W **21.9773 (5.31486)^c^** | APA was more effective than CO2 and Er,Cr:YSGG.  CO2 4-W and Er,Cr:YSGG 3-W can be regarded as surface treatment options for a  better adhesion. |
| Akin et al.  2015  **[52]** | - Control (C)  - APA Al_2_O_3_ (APA)  - Silica Coating (SC)  - Nd:YAG 0mm/1W/100mJ/20s  - Er:YAG 10mm/1.5W/150mJ/20s | 24 | zirconia | Resin composite (Variolink N) |  | 0.5 | - C **11.7 (1.83)^a^**  - APA **15.03 (1.85)^b^**  - SC **19.08 (2.97)^d^**  - Nd:YAG **16.01 (1.92)^bc^**  - Er:YAG **18.08 (3.61)^cd^** | All treatments were effective for a durable bond between zirconia and resin cement.  Lasers could be used for improving bonding between resin cement and zirconia. |
| Akpinar et al.  2015  **[67]** | -FS 90º 750mW/11cm  -FS 75º 750mW/11cm  -FS 60º 750mW/11cm  -FS 45º 750mW/11cm | 10 | zirconia | Resin cement  RelyX | Kept in desiccator/room temp/24h | 1 | -FS 90º **10.79 (1.8)^a^**  -FS 75º **13.48 (1.2)^b^**  -FS 60º **15.85 (0.81)^c^**  -FS 45º **18.2 (1.43)^d^** | As the angle between surface and laser beam decreases,  the SBS of the resin cement to zirconia material increases. |
| Akpinar, et al.  2015  **[69]** | -FS square-shaped recessed (sqR)  -FS square-shaped projection(sqP)  -FS circular-shaped recessed (cR)  -FS circular-shaped projection (cP)  *ALL: 750mW/11cm | 10 | zirconia | Resin cement  RelyX | Kept in desiccator/room temp/24h | 1 | -FS sqR **14.23 (2.98)^a^**  -FS sqP **18.26 (1.41)^b^**  -FS cR **9.86 (1.83)^c^**  -FS cP **18.62 (1.66)^d^** | SBS values of cP and sqP are greater than that of sqR and cR.  SBS values of cP are similar to sqP. |
| Gomes et al. 2015  **[10]** | - Control (C)  - Silica coating + silanization (ROC)  - Er:YAG 5mm/200mJ (LA)  - Er:YAG 5mm/200mJ + ROC (LAROC) | 15 | zirconia | - (BIF) BiFix  - (CL) Clearfil | Distilled water 37º/24h  n/2:  (TC) Thermocycled for 5000 cycles between 5-55º/30sec | 0.5 | - C+BIF+24h **7.5 (5.6)^Ba^**  - C+BIF+TC **0.0 (0.0)^Ab^**  - C+CL+24h **6.8 (3.4)^Ca^**  - C+CL+TC **1.5(2.6)^Cb^**  - ROC+BIF+24h **17.3 (6.6)^Aa^**  - ROC+BIF+TC **1.9 (1.5)^Ab^**  - ROC+CL+24h **15.8 (4.8)^Aa^**  - ROC+CL+TC **15.3 (5.8)^Aa^**  - LA+BIF+24h **5.7 (2.3)^Ba^**  - LA+BIF+TC **1.8 (3.8)^Ab^**  - LA+CL+24h **6.9 (2.0)^Ca^**  - LA+CL+TC **0.0 (0.0)^Cb^**  - LAROC+BIF+24h **18.9 (4.6)^Aa^**  - LAROC+BIF+TC **0.9(2.2)^Ac^**  -LAROC+CL+24h **11.1 (3.8)^Bb^**  -LAROC+CL+TC **9.9 (3.5)^Bb^** | Thermocycling does not affect μSBS of the resin–zirconia interface when applying a selfadhesive resin cement with 10-MDP in its composition over a zirconia surface pretreated with silica coating with or without Er:YAG.  The adhesive effectiveness is higher if the surface is only conditioned with silica coating (not applying the laser) despite the artificial aging process.  Er:YAG etching is not effective in increasing bond strength of resin-zirconia. |
| Kara et al.  2015  **[53]** | - FS Laser 11cm/400mW  - Nd:YAG 1mm/2W/100mJ  - Er:YAG 1mm/6W/300mJ | 12 | - (Z) Zirconia Z  - (ZP) Zirconia ZP | Resin cement (RelyX) | Distilled water 37º/24h  Thermocycled for 5000 cycles between 5-55º/ 30sec | 1 | - Z+FS **52.82 (13.48)**  - Z+Er:YAG **42.51 (9.59)**  - Z+ Nd:YAG **40.33 (16.59)**  - ZP+FS **51.18 (3.81)^a^**  - ZP+Er:YAG **41.77 (9.54)^b^**  - ZP+Nd:YAG **40.27 (6.00)^b^** | FS laser treatment produced the highest MPa of the processes assessed.  FS laser appears to be an effective method for bonding  resin cement to zirconia. |
| Kasraei et al  2015  **[39]** | - Control (C)  - CO_2_ 1mm/3W/10sec  - Nd:YAG 1mm/2W/10sec | 15 | zirconia | Composite resin (Filtek Z250) | - Distilled water 37º/24h  - TC:  Thermocycled for 3000 cycles/ 5-55º/ 30sec/37º/6months | 0.5 | - C **23.35 (3.12)**  - CO_2_ **14.00 (1.96)**  - Nd:YAG **18.95 (3.46)**  - C+TC **1.80(1.23)**  - CO_2_+TC **3.70 (1.56)^A^**  - Nd:YAG+TC **4.77 (1.72)^A^** | CO2 and Nd:YAG laser treatments result in an increase in durability of resin cement bond to zirconia ceramic after thermocycling and water-storage for six months. |
| Kirmali et al. 2015  **[48]** | - Control (C)  - APA CoJet (Al_2_O_3_+silica)  - Nd:YAG 1mm/1W/200mJ/20s  - Er,Cr:YSGG 10mm/1.5W/20s  - APA+Nd:YAG  -APA+Er,Cr:YSGG | 10 | zirconia | Composite resin (Grandio, Voco) | Distilled water 37º/24h | 1 | - C **15.90 (0.96)^a^**  - APA **16.73 (1.99)^ab^**  - Nd:YAG **18.87 (1.84)^b^**  - Er,Cr:YSGG **16.55 (2.38)^ab^**  - APA+Nd:YAG **17.44 (1.95)^ab^**  - APA+Er,Cr:YSGG **18.83 (2.24)^b^** | Various treatment methods of zirconia surfaces, especially Er,Cr:YSGG, improve the ceramic-composite bond strength. |
| Liu et al. 2015  **[71]** | - Control (C)  - APA Al_2_O_3_  - Nd:YAG 1W/10Hz/100mJ/30s (L1)  - Nd:YAG 1W/10Hz/100mJ/60s (L2)  - Nd:YAG 1W/10Hz/100mJ/90s (L3)  - Nd:YAG 2W/20Hz/100mJ/30s (L4)  - Nd:YAG 2W/20Hz/100mJ/60s (L5)  - Nd:YAG 2W/20Hz/100mJ/90s (L6)  - Nd:YAG 3W/30Hz/100mJ/30s (L7)  - Nd:YAG 3W/30Hz/100mJ/60s (L8)  - Nd:YAG 3W/30Hz/100mJ/90s (L9) | 20 | zirconia | Resin cement (Clearfil) | - Distilled water 37º/24h  - n/2: (TC) Thermocycled for 20000 cycles between 5-55º/28 days | 1 | - C **3.87 (1.17)^a^**  - APA **12.03 (2.58)^b^**  - L1 **4.10 (1.20)^ac^**  - L2 **4.51 (1.2)^ac^**  - L3 **4.41 (1.16)^ac^**  - L4 **5.00 (1.53)^ac^**  - L5 **4.47 (1.31)^ac^**  - L6 **4.62 (0.90)^ac^**  - L7 **5.40 (1.68)^ac^**  - L8 **6.33 (1.69)^c^**  - L9 **5.81 (1.89)^ac^**  - C+TC **3.81 (1.07)^a^**  - APA+TC **12.16 (3.39)^b^**  - L1+TC **3.79 (1.01)^a^**  - L2+TC **3.98 (1.10)^a^**  - L3+TC **4.45 (1.23)^a^**  - L4+TC **4.52 (1.19)^a^**  - L5+TC **4.58 (1.28)^a^**  - L6+TC **4.24 (0.89)^a^**  - L7+TC **5.29 (1.42)^a^**  - L8+TC **5.73 (1.67)^a^**  - L9+TC **5.45 (1.38)^a^** | Nd: YAG can change morphological characteristics of dental zirconia ceramics and roughen the ceramic surface, but cannot increase the SBS of the ceramics to resin cement.  Enhancing output power and extending irradiation time  cannot induce higher bond strength of zirconia and might cause material defect.  Air abrasion with alumina particles can improve bonding property of dental zirconia ceramics to resin cement. |
| Loffredo et al.  2015  **[54]** | - 10%HF acid (HF)  - APA Al_2_O_3_ + HF (APAHF)  - Er:YAG 500mJ/4Hz  - Er:YAG 500mJ/4Hz + HF  - Er:YAG 400mJ/6Hz  - Er:YAG 400mJ/6Hz + HF    *Er:YAG 0.3mm/40s | 7 | feldspathic | Silane + resin cement (Rely X ARC) | Distilled water 37º/24h | 0.5 | - HF **17.55 (3.9)^bcd^**  - APAHF **18.8 (2.6)^cd^**  - Er:YAG500 **21.8 (2.5)^d^**  - Er:YAG500+HF **12.62 (3.5)^ab^**  - Er:YAG400 **15.81 (4.3)^abc^**  - Er:YAG400+HF **11.59 (3.4)^a^** | Er:YAG at 500 mJ/4 Hz obtained results similar to the HF acid etching and APAHF groups, and was superior to the other Er:YAG laser groups. |
| Sadeghi et al. 2015  **[31]** | - Control (C)  - 9% HF acid (HF)  - Er:YAG 2W/100mJ/1mm/20s  - Er:YAG 3W/150mJ/1mm/20s  - Er:YAG 4W/200mJ/1mm/20s  - Er:YAG 5W/250mJ/1mm/20s | 12 | feldspathic | - Silane + resin composite (Point 4, Kerr) | Distilled water 37º/2 weeks  Thermocycled for 1500 cycles between 5-55º/60sec | 1 | - C **3.76 (0.89)**  - HF **12.29 (3.04)**  - Er:YAG 2W **1.96 (0.76)**  - Er:YAG 3W **2.23 (0.60)**  - Er:YAG 4W **2.93 (0.47)**  - Er:YAG 5W **8.00 (2.22)** | HF acid created a higher SBS than Er:YAG laser with different power outputs.  Er:YAG at 5W, 250 mJ/20 Hz  was the most effective of the laser groups. |
| Taniş and Akçaboy  2015  **[11]** | - APA Al_2_O_3_  - APA+Silica Coating (SC)  - APA+Nd:YAG 2W/100mJ/2min | 10 | zirconia | Resin cements:  - (P) Panavia F  - (V) Variolink II | Distilled water 37º  - 24h  - 14 days | 1 | - APA+P+24h **5.97 (2.27)^Aa^**  - SC+P+24h **12.13 (4.69)^Ba^**  - Nd:YAG+P+24h **6.32 (3.34)^Aa^**  - APA+P+14d **4.35 (3.11)^Aa^**  - SC+P+14d **10.77 (4.20)^Ba^**  - Nd:YAG+P+14d **4.85 (1.90)^Aa^**  - APA+V+24h **2.24 (1.05)^Ab^**  - SC+V+24h **15.05 (11.53)^Ba^**  - Nd:YAG+V+24h **2.97 (0.80)^Cb^**  - APA+V+14d **1.98 (1.54)^Ab^**  - SC+V+14d **14.30 (5.56)^Ba^**  - Nd:YAG+V+14d **2.51 (1.65)^Aa^** | MDP containing resin cements increase the bond strength of sandblasted zirconia and tribochemical silica coating following sandblasting is an effective method for achiev­ing resin bonding of zirconia ceramics. But long-term performance of tribochemical silica coating method is questionable. |
| Yavuz et al.  2015  **[55]** | - Control (C)  - APA Al_2_O_3_ (APA)  - Silica coating (SC)  - 5% HF acid (HF)  - Er:YAG 1mm/10W/500mJ  - Nd:YAG 1mm/2W/100mJ  - FS laser 750mW (FS) | 12 | - Lithium disilicate (L)  - Zirconia (Z) | Silane + composite resin | Distilled water 37º/24h  Thermocycled for 1000 cycles between 5-55º/30sec | 0.5 | - L+C **7.61 (3.69)^cdA^**  - L+APA **9.80 (3.65)^bcA^**  - L+SC **29.10 (4.01)^aA^**  - L+HF **26.07 (5.84)^aA^**  - L+Er:YAG **4.92 (2.53)^dA^**  - L+Nd:YAG **5.30 (2.13)^dA^**  - L+FS **12.66 (1.55)^bA^**  - Z+C **7.62 (4.26)^cA^**  - Z+APA **19.94 (5.04)^bB^**  - Z+SC **28.08 (5.30)^aA^**  - Z+HF **16.72 (3.39)^bB^**  - Z+Er:YAG **9.47 (3.47)^cA^**  - Z+Nd:YAG **9.08 (2.97)^cA^**  - Z+FS **15.12 (2.67)^bA^** | Silanization after silica coating improves the bond  strengths to both porcelain surfaces, while HF etching  is similarly favorable only for lithium disilicate surfaces.  Treatment with FS laser seems to be a suitable conditioning  method to strengthen the bonding of resin cements  to ceramic surfaces. |
| Arami et al.  2014  **[56]** | - Control (C)  - APA Al_2_O_3_ (APA)  - Er:YAG 4mm/2W/10s  - Nd:YAG 1mm/1.5W/2min | 10 | zirconia | Composite resin (Clearfil) |  | 0.5 | - C **11.87 (2.97)^a^**  - APA **17.24 (3.22)^b^**  - Er:YAG **11.15 (1.36)^a^**  - Nd:YAG **9.32 (1.65)^a^** | APA is the most effective method for zirconia surface treatment.  Nd:YAG laser resulted in decreased SBS. |
| Erdem et al.  2014  **[57]** | - Control (C)  - APA Al_2_O_3_ (APA)  - APA + silica coating + silane (SC)  - Er:YAG 10mm/2W/200mJ/10s | 10 | zirconia | Resin cement  - Panavia F (P)  - Rely X U100 (R)  - Clearfil (CL)  - Superbond (SB)  - Multilink (M) | Distilled water 37º/60 days | 0.5 | - C+P **0.2^a^**  - APA+P **12.2^b1^**  - SC+P **13.3^b1^**  - Er:YAG+P **0.4^a^**  - C+R **0.3^a^**  - APA+R **12.7^b1^**  - SC+R **10.5^c2^**  - Er:YAG+R **0.1^a^**  - C+CL **0.0^a^**  - APA+CL **1.8^b2^**  - SC+CL **8.8^c2^**  - Er:YAG+CL **0.1^a^**  - C+SB **0.2^a^**  - APA+SB **0.4^a2^**  - SC+SB **0.9^a3^**  - Er:YAG+SB **0.4^a^**  - C+M **0.0^a^**  - APA+M **0.3^a2^**  - SC+M **10.1^b12^**  - Er:YAG+M **0.0^a^** | Bond strengths of resin cements in untreated control and laser-applied specimens were low.  APA and SC methods can improve SBS  PMC adhesive resin  cements (Panavia 2.0, Clearfil Esthetic, and Rely  X U100) in combination with APA or SC application  produced higher bond strength.  SC system increased bond strength values of phosphate  monomer–free adhesive resin cement (Multilink).  Regardless of the dual and self-cure resin cement used, laser treatment did not improve resin bond strength. |
| Ghasemi et al. 2014  **[49]** | - Control (C)  - AS2: Er,Cr:YSGG 1mm/2W/50sec (after sintering)  - AS3: Er,Cr:YSGG 1mm/3W/50sec (after sintering)  - PS2: Er,Cr:YSGG 1mm/2W/50sec (pre-sintered)  - PS3: Er,Cr:YSGG 1mm/3W/50sec (pre-sintered)  - APA Al_2_O_3_ | 15 | zirconia | Panavia 2.0 | 98% humidity/ 37º/48h | 0.5 | - C **26.86 (6.11)^a^**  - AS2 **32.04 (7.37)^ab^**  - AS3 **34.92 (7.70)^b^**  - PS2 **30.38 (6.34)^ab^**  - PS3 **29.51 (5.32)^ab^**  - APA **52.55 (9.23)** | APA is more effective than Er,Cr:YSGG.  Er,Cr:YSGG at 3W after sintering can be an addequate conditioning technique for a better bond strength |
| Kasraei, et al. 2014  **[40]** | - Control (C)  - CO_2_ 1mm/3W/10s | 15 | zirconia | Composite resin (Filtek Z 250) | Distilled water 37º/24h | 0.5 | - C **5.95 (1.14)**  - CO_2_ **12.12 (3.02)** | CO_2_ laser enhances primary bond strength between resin  cement and zirconia ceramic. |
| Kasraei et al. 2014  **[28]** | - Control (C)  - CO_2_ 1mm/3W/265.39J/cm^2^/10s  - Er:YAG 0.5mm/2W/200mJ/10s | 15 | zirconia | Composite resin (Filtek Z 250) | Distilled water 37º/24h | 0.5 | - C **5.97 (1.14)^a^**  - CO_2_  **12.12 (3.02)^c^**  - Er:YAG **8.65 (1.77)^b^** | CO2 and Er:YAG increased SBS of resin cement to zirconia ceramic, CO_2_ being superior |
| Subaşi and İnan  2014  **[58]** | - Control (C)  - APA Al_2_O_3_  - Silica Coating (SC)  - Er:YAG 1mm/4W/400mJ/15s  - APA+Er:YAG | 10 | zirconia | Composite resin cilinders  cemented with:  - (R) RelyX  - (CL) Clearfill  - (P) Panavia F | Distilled water 37º/24h  Thermocycled for 6000 cycles between 5-55º/30sec | 0.5 | - C+R **2.43 (0.62)^b,1^**  - APA+R **3.07 (0.55)^c,1^**  - SC+R **2.59 (0.35)^bc,1^**  - Er:YAG+R **1.76 (0.37)^a,1^**  - APA+Er:YAG+R **2.72 (0.77)^bc,1^**  - C+CL **3.52 (0.39)^ab,3^**  - APA+CL **3.47 (1.04)^ab,12^**  - SC+CL **3.09 (0.95)^a,12^**  - Er:YAG+CL **2.79 (0.64)^a,2^**  - APA+Er:YAG+CL **4.11 (0.68)^b,2^**  - C+P **2.95 (0.23)^a,2^**  - APA+P **4.00 (0.26)^b,2^**  - SC+P **3.74 (0.89)^b,2^**  - Er:YAG+P **2.48 (0.38)^a,2^**  - APA+Er:YAG+P **4.18 (0.58)^b,2^** | All surface treatment methods except laser were suitable for use on the zirconia surface before cementation.  Surface treatment or cement selection could affect the bond  strength between the resin cement and the zirconia. Cement selection was more important than surface  treatment, and PMC cements  (Cl and P) were suitable for cementing zirconia. |
| Kursoglu et al.  2013  **[50]** | - Control (C)  - 9.5% HF acid (HF)  - Er,Cr:YSGG 1.5W  - Er,Cr:YSGG 2.5W  - Er,Cr:YSGG 6W  *Er,Cr:YSGG 1mm/300mJ/60s | 10 | Lithium disilicate | Composite resin (Filtek) + resin cement (RelyX) | Distilled water 37º/24h | 1 | - C **1.95 (1.06)^c^**  - HF **8.42 (1.86)^a^**  - Er,Cr:YSGG 1.5W **3.88 (1.94)^b^**  - Er,Cr:YSGG 2.5W **3.65 (1.87)^b^**  - Er,Cr:YSGG 6W **3.59 (1.19)^c^** | Er,Cr:YSGG at 1.5 and 2.5 W increases SBS compared with untreated surfaces.  HF acid increased SBS more effectively than any laser treatment. |
| Lin et al. 2013  **[59]** | - Control (C)  - APA Al_2_O_3_  - Er:YAG 1W/100mJ/5s (L1)  - Er:YAG 1W/100mJ/10s (L2)  - Er:YAG 1W/100mJ/15s (L3)  - Er:YAG 2W/200mJ/5s (L4)  - Er:YAG 2W/200mJ/10s (L5)  - Er:YAG 2W/200mJ/15s (L6)  - Er:YAG 3W/300mJ/5s (L7)  - Er:YAG 3W/300mJ/10s (L8)  - Er:YAG 3W/300mJ/15s (L9) | 10 | zirconia | Resin cement (Clearfil) | Distilled water 37º/24h  n/2: (TC) Thermocycled for 20000 cycles between 5-55º/28 days | 1 | - C **3.87 (1.17)^a^**  - APA **12.03 (2.58)^b^**  - L1 **4.26 (0.90)^a^**  - L2 **3.66 (1.03)^a^**  - L3 **3.83 (0.88)^a^**  - L4 **4.60 (1.57)^a^**  - L5 **5.37 (1.37)^a^**  - L6 **4.82 (1.46)^a^**  - L7 **4.88 (1.73)^a^**  - L8 **5.74 (1.86)^a^**  - L9 **5.61 (1.47)^a^**  - C+TC **3.81 (1.07)^a^**  - APA+TC **12.16 (3.38)^b^**  - L1+TC **3.90 (1.11)^a^**  - L2+TC **3.26 (0.90)^a^**  - L3+TC **4.09 (1.28)^a^**  - L4+TC **4.30 (1.47)^a^**  - L5+TC **4.26 (1.07)^a^**  - L6+TC **4.19 (1.28)^a^**  - L7+TC **4.58 (1.47)^a^**  - L8+TC **5.41 (1.46)^a^**  - L9+TC **5.52 (1.51)^a^** | Er:YAG laser irradiation can roughen the surface of zirconia ceramics, but cannot increase the SBS of the ceramics to resin cement.  Enhancing irradiation intensities and extending irradiation time cannot induce higher SBS of zirconia ceramics,  and may cause material defect. APA can improve bonding property of zirconia ceramics to resin cement |
| Saraç et al.  2013  **[41]** | - Control (C)  - 4% HF acid (HF)  - Er:YAG 2W/200mJ/2s  - CO_2_ 3W  - APA Al_2_O_3_ (APA)  - Silica coating (SC) | 15 | feldspathic | Silane + composite resin (Reflections) | Distilled water 37º/24h  Thermocycled for 500 cycles between 5-55º/30sec | 1 | - C **5.56 (0.40)**  - HF **13.71 (0.48)^a^**  - Er:YAG **10.73 (0.90)**  - CO_2_ **12.99 (1.06)^a^**  - APA **16.88 (1.18)^b^**  - SC **17.73 (1.50)^b^** | Surface treatments affected SBS. SC and APA are the most effective treatments showing higher SBS.  CO_2_ laser was more effective than Er:YAG |
| Usumez et al.  2013  **[72]** | - Control (C)  - Feldspathic glazing + 9.5 HFA (G)  - APA Al_2_O_3_ (APA)  - Nd:YAG 180μs pulse (ND180)  - Nd:YAG 320μs pulse (ND320)  * Nd:YAG 200mJ/60s | 15 | zirconia | Resin Cement (Clearfil) |  | 1 | - C **3.73 (1.9)^a^**  - G **4.99 (1.7)^a^**  - APA **4.26 (1.7)^a^**  - ND180 **8.17 (1.9)^b^**  - ND320 **6.99 (1.6)^b^** | SBS of resin cement  to the zirconia surface in the laser groups was comparable to  each other and was significantly higher than the other groups. |
| Yavuz et al.  2013  **[60]** | - APA Al_2_O_3_ (APA)  - 5% HF acid (HF)  - Er:YAG 1mm/10W/500mJ/20s  - APA+Er:YAG  - HF+Er:YAG | 10 | - (L) Lithium disilicate IPS Empress 2  - (F) Feldspathic VITA VM 9 | Silane + resin cement (Panavia F) | Distilled water room temp/24h | 0.5 | - L+APA **6.10 (2.16)^ab^**  - L+HF **6.40 (1.72)^ab^**  - L+Er:YAG **4.47 (2.48)^b^**  - L+APA+Er:YAG **7.99 (3.48)^a^**  - L+HF+Er:YAG **4.20 (1.61)^b^**  - F+APA **6.22 (2.57)^ab^**  - F+HF **5.61 (1.28)^ab^**  - F+Er:YAG **4.86 (1.36)^ab^**  - F+APA+Er:YAG **6.61 (2.56)^ab^**  - F+HF+Er:YAG **3.96 (1.53)^b^** | SBS after surface treatments varied in accordance with the type of ceramic.  For lithium disilicate, SBS was significantly different between laser groups.  No significant difference was found for feldspathic ceramic.  HF+Er:YAG and Er:YAG groups showed low SBS values |
| Liu et al.  2012  **[61]** | - Control (C)  - Grinding (G)  - APA Al_2_O_3_ (APA)  - Er:YAG 200mJ/5s | 5 | zirconia | Resin composite (Ceram.X) | Distilled water 37º/24h | 1 | - C **16.4 (1.2)**  - G **17.7 (1.9)**  - APA **21.9 (1.3)**  - Er:YAG **31.5 (1.3)** | SBS of laser irradiated and sandblasted zirconia are better than those treated by hand grinding |
| Tarcin et al.  2012  **[62]** | - Control (C)  - APA Al_2_O_3_ (APA)  - Er:YAG 1mm/2W/200mJ | 10 | feldspathic | - Silane + self-etch adhesive + resin composite (SEA)  - 36% OP acid + silane + Total-etch adhesive + composite resin (TEA) | Distilled water 37º/24h | 1.5 | - C+SEA **9.79 (2.55)**  - C+TEA **5.72 (0.98)**  - APA+SEA **11.99 (3.30)**  - APA+TEA **9.53 (4.26)**  - Er:YAG+SEA **12.05 (1.85)**  - Er:YAG+TEA **7.48 (1.33)** | A selfetch adhesive application after Er:YAG laser treatment could be the method of choice when reparing porcelain restorations. |
| Ural et al.  2012  **[42]** | - Control (C)  - CO_2_ 2W  - CO_2_ 3W  - CO_2_ 4W  - CO_2_ 5W | 10 | zirconia | Composite resin (Filtek) + resin cement (Panavia F) | Distilled water 37º/24h | 1 | - C **13.4 (3.1)^a^**  - CO_2_ 2W **21.0 (2.7)^c^**  - CO_2_ 3W **20.9 (3.8)^c^**  - CO_2_ 4W **17.2 (2.1)^b^**  - CO_2_ 5W **14.4 (1.6)^ab^** | There might be a relationship between laser output power and SBS for zirconia ceramics. CO_2_ laser may be effective for improving SBS. |
| Yucel et al.  2012  **[73]** | - Control (C)  - APA Al_2_O_3_ (APA)  - 4.9% HF acid (HF)  - APA+HF  - Nd:YAG 1mm/2W/100mJ  - Nd:YAG+HF | 10 | Lithium disilicate  - IPS e.max (EM)  - IPS Empress (E) | Resin cement (Variolink II) | Thermocycled for 2000 cycles between 5-55º/25sec | 0.5 | - EM+C **7.5 (1.4)^a^**  - EM+APA **17.7 (0.9)^b^**  - EM+HF **22.1 (3.4)^c^**  - EM+APA+HF **28.2 (1.9)^d^**  - EM+Nd:YAG **11.9 (0.9)^e^**  - EM+Nd:YAG+HF **23.7 (2.2)^c^**  - E+C **12.6 (1.8)^a^**  - E+APA **20.5 (1.3)^b^**  - E+HF **25.9 (2.1)^c^**  - E+APA+HF **30.2 (2.0)^d^**  - E+Nd:YAG **15.6 (2.0)^e^**  - E+Nd:YAG+HF **27.4 (3.1)^c^** | IPS Empress 2 ceramic showed significantly higher SBS values than IPS e.max  SBS were significantly affected by surface treatments.  The micromorphology of ceramic surfaces after Nd:YAG laser irradiation was similar to the untreated surfaces. |
| Akyil et al. 2011  **[63]** | -Control (C)  -HF  -APA Al_2_O_3_  -Er:YAG 1mm/4W/400mJ/1min  -Nd:YAG 1mm/2W/200mJ/1min  - APA Al_2_O_3_+HF  -Er:YAG+HF  -Nd:YAG+HF | 32/36/33/18/20/29/26/24 | feldspathic | Silane + Resin cement  Panavia 2.0 | Distilled water 37º/24h  Thermocycled for 1000 cycles between 5-55º/  30sec | 0.1 | -C **8.02 (2)^bc^**  -HF **17.01 (4.5)^f^**  -APA **14.58 (3.21)^e^**  -Er:YAG **5.28 (0.74)^a^**  -Nd:YAG **6.51 (1.74)^ab^**  - APA+HF **9.08 (3.34)^c^**  -Er:YAG+HF **12.25 (4.23)^d^**  -Nd:YAG+HF **11.73 (5.22)^d^** | HF showed the best SBS  APA, APA+HF and lasers + HF  promoted an improvement in adhesion between Panavia F and feldspathic ceramic.  Er:YAG or Nd:YAG lasers used alone negatively changed the bonding of the resin cement. |
| Foxton et al. 2011  **[64]** | - Control (C)  - APA Al_2_O_3_ (APA)  - Er:YAG 0mm/200mJ/5sec | 0/4/2/2/7/4/5/5/2/2/3/4/2/8/7/5/8/3/8/8/7/5/9/4 | - (A) Aluminum Oxide  - (Z) Zirconia | - (V) Variolink II  - (N) NAC 100 | water storage 37º  - 24 hours  - 6 months | 1 | A+V+1day+C n=0  A+V+1day+APA **17.63 (4.29)**  A+V+1day+Er:YAG **15.15 (0.21)**  A+V+6m+C **11.00 (0.00)**  A+V+6m+APA **9.01 (1.18)**  A+V+6m+Er:YAG **10.35 (2.32)**  Z+V+1day+C **18.94 (2.95)**  Z+V+1day+APA **20.98 (3.69)**  Z+V+1day+Er:YAG **13.95 (0.92)**  Z+V+6m+C **14.65 (0.49)**  Z+V+6m+APA **8.97 (2.76)**  Z+V+6m+Er:YAG **8.3 (1.15)**  A+N+1day+C **17.3 (0.99)**  A+N+1day+APA **17.53 (1.10)**  A+N+1day+Er:YAG **16.05 (1.33)**  A+N+6m+C **12.72 (3.92)**  A+N+6m+APA **25.68 (7.64)**  A+N+6m+Er:YAG **16.73 (8.44)**  Z+N+1d+C **19.08 (2.14)**  Z+N+1d+APA **19.55 (2.02)**  Z+N+1d+Er:YAG **16.27 (4.14)**  Z+N+6m+C **19.84 (3.82)**  Z+N+6m+APA **14.79 (2.13)**  Z+N+6m+Er:YAG **10.93 (3.08)** | Er:YAG did not result in a durable cement-ceramic bond.  In the case of aluminum oxide, Er:YAG did not improve SBS compared with the APA and control.  A durable bond to APA aluminum oxide was formed when conventional dual-cured resin cement was used in conjunction with a ceramic primer containing the  MDP; this was not the case for  zirconia.  When the aluminum oxide and zirconia specimens were left untreated, a durable bond was formed to both materials when  the surfaces were applying primer containing MDP and bonded with conventional  dual-cured resin cement. |
| Kara et al.  2011  **[74]** | - APA Al_2_O_3_ (APA)  - 5% HF acid (HF)  - Nd:YAG 1mm/2W/100mJ | 12 | feldspathic | Resin cement (Clearfil) | Distilled water room temp/24h | 1 | - APA **84.72 (2.46)**  - HF **59.33 (3)**  - Nd:YAG **58.99 (5.77)** | APA showed the highest SBS. No significant differences between Nd:YAG and HF on SBS |
| Maruo et al. 2011  **[43]** | - Control (C)  - Ceramic Primer (P)  - APA Al_2_O_3_ (APA)  - CO_2_ 6W/60s  - CO_2_ 7W/60s  - CO_2_ 8W/60s  - CO_2_ 9W/60s  - CO_2_ 10W/60s  - CO_2_ 8W/60s + APA  - CO_2_ 8W/45s  - CO_2_ 8W/90s | 10 | zirconia | Silane + Resin cement (Linkmax) | Distilled water 37º/24h | 0.5 | - C **2.21 (0.53) ^b^**  - P **1.78 (0.56)^a^**  - APA **5.39 (0.64)^c^**  - CO_2_6W **4.95 (1.54)^c^**  - CO_2_7W **5.03 (1.30)^c^**  - CO_2_8W **6.68 (1.69)^c^**  - CO_2_9W **6.46 (1.23)^c^**  - CO_2_10W **6.06 (1.26)^c^**  - CO_2_8W+APA **7.49 (3.08)^c^**  - CO_2_8W/45s **4.94 (1.24)**  - CO_2_8W/90s **5.75 (1.41)** | CO2 laser can increase the adhesive strength of a resin cement bonded to ceramic. |
| Paranhos et al.  2011  **[24]** | - Control (C)  - APA Al_2_O_3_ (APA)  - Silica coating (SC)  - C+Nd:YAG 0mm/2W/100mJ  - APA + Nd:YAG 0mm/2W/100mJ  - SC + Nd:YAG  - C+CO_2_ 1mm/5W/5J  - APA + CO_2_ 1mm/5W/5J  - SC + CO_2_ 1mm/5W/5J | 17 | zirconia | Silane + Resin cement (Panavia F) | Distilled water 24h |  | - C **4.65 (1.31)^Bc^**  - APA **8.79 (1.39)^Ab^**  - SC **7.56 (1.11)^Ac^**  - C+Nd:YAG **14.09 (1.88)^Ba^**  - APA+Nd:YAG **16.20 (1.87)^Aa^**  - SC+Nd:YAG **15.21 (1.87)^ABa^**  - C+CO_2_ **7.92 (1.97)^Bb^**  - APA + CO_2_ **6.24 (2.53)^Cc^**  - SC + CO_2_ **10.51 (3.12)^Ab^** | Nd:YAG lasers create roughness on zirconia and increase  its SBS to resin,.  SC could potentially increase the SBS of lased and nonlased zirconia.  Significant microcracks  were found on CO_2_ specimens, which contraindicates its use. |
| Akyil et al.  2010  **[44]** | -Control (C)  - APA Al_2_O_3_  -Silica coating (SC)  -Er:YAG 1mm/2W/200mJ/10sec  -Nd:YAG 1mm/2W/100mJ/2min  -CO_2_ 1mm/4W/cont/50sec  -APA+Er  -APA+Nd  -APA + CO_2_ | 16/16/16/15/15/15/16/16/16 | zirconia | Silane + Resin cement (Clearfil) | Distilled water 37º/24h  Thermocycled for 500 cycles between 5-55º/30sec | 0.5 | -C **17.02 (4.14)^ab^**  - APA Al_2_O_3_ **23.46 (2.77)^b^**  -SC **23.39 (2.40)^b^**  -Er:YAG **19.69 (6.30)^ab^**  -Nd:YAG **15.62 (5.05)^ab^**  -CO_2_ **22.35 (6.13)^ab^**  -APA+Er **14.85 (9.99)^a^**  -APA+Nd **20.82 (11.61)^ab^**  -APA + CO_2_ **19.30 (8.50)^ab^** | APA and SC are the most effective methods for improving the SBS of cement to zirconia.  Er:YAG and CO_2_ can increase the bond strength.  Nd:YAG can decrease SBS.  CO2 or Er:YAG after APA can decrease the SBS, but Nd:YAG laser irradiation can increase it. |
| Akyil et al.  2010  **[65]** | - Control (C)  - 9.5% HF acid (HF)  - Er:YAG 3W/300mJ/1min  - Nd:YAG 1W/100mJ/1min  - HF+Er:YAG  - HF+Nd:YAG | 12 | feldspathic | Silane + composite resin (Arabesk Top) | Distilled water 37º/24h  Thermocycled for 1000 cycles between 5-55º/30sec | 0.5 | - C **5.83 (1.22)^a^**  - HF **15.21 (2.54)^b^**  - Er:YAG **3.96 (0.55)^c^**  - Nd:YAG **6.08 (1.33)^a^**  - HF+Er:YAG **12.74 (2.22)^d^**  - HF+Nd:YAG **12.59 (1.95)^d^** | HF acid was the most effective  for increasing SBS.  Er:YAG showed lower SBS than the control group.  Nd:YAG showed similar results to control group  HF acid + laser produces greater SBS than laser-only. |
| Chen et al.  2010  **[45]** | - APA Al_2_O_3_ (APA)  - APA + CO_2_ 30mm (APA+CO_2_)  - APA + Primer (APA + P)  - APA + P + CO_2_ | 5 | Leucite glass | Composite resin (Lite-Fil II) | Distilled water 37º/24h  n/2:  (TC)Thermocycled for 2000 cycles between 4-60º/60sec | 1 | - APA **0.26**  - APA+CO_2_ **0.05**  - APA+P **8.2**  - APA+P+CO_2_ **9.7**  - APA+TC **FAILED**  - APA+CO_2_+TC **FAILED**  - APA+P+TC **3.6**  - APA+P+CO_2_+TC **7.7** | Silane-treated porcelain  irradiated with carbon dioxide laser, showed increased SBS, being pronouncedly higher than other surface treatments. |
| Da Silva Ferreira et al.  2010  **[66]** | - APA Al_2_O_3_ (APA)  - APA+Er:YAG 500mJ/20s  - APA+Nd:YAG 1mm/1W/100mJ | 20 | feldspathic | Silane +  - (ARC) Resin cement (RelyX ARC)  - (U100) Self adhesive resin cement (RelyX U100) | Distilled water 37º/24h | 0.5 | - APA+ARC **10.35 (2.37)**  - APA+Er:YAG+ARC **11.12 (2.20)**  - APA+Nd:YAG+ARC **9.78 (2.40)**  - APA+U100 **10.71 (3.17)**  - APA+Er:YAG+U100 **11.18 (3.61)**  - APA+Nd:YAG+U100 **9.71 (2.09)** | APA associated with Er:YAG or Nd:YAG laser and self-adhesive cement can be an alternative technique to bond to feldspathic ceramic, as it was as effective as the conventional treatment with APA and HF acid using  the conventional resin cement. |
| Ural et al.  2010  **[46]** | - Control (C)  - APA  - HF acid  - CO_2_ 3W | 10 | zirconia | Composite resin (Filtek) + resin cement (Panavia F) | Distilled water 37º/24h | 1 | - C **13.4 (3.1)^a^**  - APA **16.4 (3.4)^a^**  - HF **14.1 (4.7)^a^**  - CO_2_ **20.9 (3.7)^b^** | CO2 laser may represent an  effective method for conditioning zirconia surfaces, improving SBS. |
| Spohr et al.  2008  **[76]** | - APA Al_2_O_3_ (APA)  - APA + silica coating (SC)  - APA + Nd:YAG 1mm/2W/100mJ/2min | 20 | zirconia | Resin cement (Panavia Fluoro) + composite resin (Filtek) | Distilled water 37º/24h | 0.5 | - APA **11.81 (3.12)^a^**  - SC **15.75 (4.45)^b^**  - APA+Nd:YAG **18.70 (5.14)^c^** | Nd:YAG was an effective surface treatment for bonding between Zirconia and Panavia Fluoro Cement. |
| Shiu et al. 2007  **[67]** | - Control (C)  - 10% HF acid 2 min (HF)  - 37% HPO acid 1 min (HPO)  - 1.23% APF acid 10 min (APF)  - Diamond bur (DB)  - APA Al_2_O_3_ (APA)  - APA+HF  - Silica coating (SC)  - Er:YAG 500mJ/2 min  - APA+Er:YAG | 10 | feldspathic | Silane coating + resin cement (Rely X) | Distilled water 37º/24h | 0.5 | - C **4.15 (2.10)**  - HF **16.80 (5.15)**  - HPO **3.08 (1.49)**  - APF **5.41 (1.47)**  - DB **9.66 (2.51)**  - APA **16.22 (4.28)**  - APA+HF **9.88 (2.76)**  - SC **15.14 (2.65)**  - Er:YAG **3.67 (2.18)**  - APA+Er:YAG **9.53 (2.22)** | HF, SC, and APA were the most effective surface treatments and produced the strongest bonds.  Er:YAG laser resulted in low bond strength, and thus appears to be inadequate  for clinical use within the parameters tested here. |
| Da Silveira et al.  2005  **[75]** | - APA Al_2_O_3_ (APA)  - APA + silica coating (SC)  - APA+Nd:YAG 1mm/2W/100mJ | 15 | alumina | Silane + resin cement (Panavia Fluoro) + composite resin (Filtek) | Distilled water 37º/24h | 0.5 | - APA **14.38 (2.97)^a^**  - APA+SC **20.09 (3.46)^b^**  - APA+Nd:YAG **23.21 (3.72)^c^** | Nd:YAG was the most effective surface treatment for bonding between Alumina and Panavia Fluoro Cement. |
