# Supplementary material for: The effects of lasers on bond strength to ceramic materials: A systematic review and meta-analysis
Source: PLoS One. 2018 Jan 2;13(1):e0190736. doi: 10.1371/journal.pone.0190736 (PMC5749860; doi:10.1371/journal.pone.0190736)
Supplement: S2 Table — (DOCX) [file pone.0190736.s002.docx]

Risk of bias assessment.

| AUTHOR/Year | Sample size calculation | Adequate control group | Laser Settings | Materials used according to manufacturers instructions | Surface treatment by single operator | Bonding by single operator | Adequate statistical analysis | Risk of Bias |
| --- | --- | --- | --- | --- | --- | --- | --- | --- |
| El Gamal et al. 2017 [21] | N | Y | Y | Y | N | N | Y | Medium |
| Zanatta et al. 2017 [70] | N | Y | Y | Y | N | N | Y | Medium |
| Ahrari et al. 2016 [38] | N | Y | Y | Y | N | N | Y | Medium |
| Aras et al. 2016 [47] | N | Y | Y | Y | N | N | Y | Medium |
| Barutcigil et al. 2016 [22] | N | Y | Y | Y | N | N | Y | Medium |
| Dede et al. 2016 [37] | N | Y | Y | Y | N | N | Y | Medium |
| Esteves-Oliveira et al. 2016 [29] | N | N | Y | Y | N | N | N | High |
| Uzun et al. 2016 [9] | N | N | Y | N | N | N | Y | High |
| Vicente et al. 2016 [25] | N | Y | Y | N | N | N | Y | High |
| Vicente Prieto et al. 2016 [26] | N | Y | Y | Y | N | N | Y | Medium |
| Yenisey et al. 2016 [51] | N | Y | Y | Y | N | N | Y | Medium |
| Akhavan Zanjani et al. 2015 [30] | N | N | Y | Y | N | N | Y | High |
| Akin et al. 2015 [52] | N | Y | Y | Y | Y | N | Y | Medium |
| Akpinar, et al. 2015 [68] | N | N | Y | N | N | N | Y | High |
| Akpinar et al. 2015 [69] | N | N | Y | N | N | N | Y | High |
| Gomes et al. 2015 [10] | N | Y | Y | Y | N | N | Y | Medium |
| Kara et al. 2015 [53] | N | N | Y | Y | N | N | Y | High |
| Kasraei et al. 2015 [39] | N | Y | Y | Y | N | N | Y | Medium |
| Kirmali et al. 2015 [48] | N | Y | Y | Y | N | N | Y | Medium |
| Liu et al. 2015 [71] | N | Y | Y | Y | N | Y | Y | Medium |
| Loffredo et al. 2015 [54] | N | N | Y | Y | Y | N | Y | Medium |
| Sadeghi et al. 2015 [31] | N | Y | Y | N | N | N | Y | High |
| Taniş and Akçaboy 2015 [11] | N | N | Y | Y | N | N | Y | High |
| Yavuz et al. 2015 [55] | N | Y | Y | Y | N | N | Y | Medium |
| Arami et al. 2014 [56] | N | Y | Y | Y | N | N | Y | Medium |
| Erdem et al. 2014 [57] | N | Y | Y | Y | Y | Y | N | Medium |
| Ghasemi et al. 2014 [49] | N | Y | Y | Y | N | N | Y | Medium |
| Kasraei et al. 2014 [40] | N | Y | Y | Y | N | N | Y | Medium |
| Kasraei et al. 2014 [32] | N | Y | Y | N | N | N | Y | High |
| Subaşi and İnan 2014 [58] | N | Y | Y | Y | N | N | Y | Medium |
| Kursoglu et al. 2013 [50] | N | Y | Y | Y | N | Y | Y | Medium |
| Lin et al. 2013 [59] | N | Y | Y | Y | N | N | Y | Medium |
| Saraç et al. 2013 [41] | N | Y | Y | N | N | N | Y | High |
| Usumez et al. 2013 [72] | N | Y | Y | Y | N | N | Y | Medium |
| Yavuz et al. 2013 [60] | N | N | Y | Y | Y | N | Y | Medium |
| Liu et al. 2012 [61] | N | Y | Y | N | N | N | Y | High |
| Tarcin et al. 2012 [62] | N | Y | Y | Y | Y | Y | Y | Low |
| Ural et al. 2012 [42] | N | Y | Y | N | N | N | Y | High |
| Yucel et al. 2012 [73] | N | Y | Y | Y | N | N | Y | Medium |
| Akyil et al. 2011 [63] | N | Y | Y | Y | N | N | Y | Medium |
| Foxton et al. 2011 [64] | N | Y | Y | Y | N | N | N | High |
| Kara et al. 2011 [74] | N | N | Y | Y | N | N | Y | High |
| Maruo et al. 2011 [43] | N | Y | Y | N | N | N | Y | High |
| Paranhos et al. 2011 [24] | N | Y | Y | Y | N | N | Y | Medium |
| Akyil et al. 2010 [44] | N | Y | Y | Y | N | N | Y | Medium |
| Akyil et al. 2010 [65] | N | Y | Y | Y | N | N | Y | Medium |
| Chen et al. 2010 [45] | N | N | Y | N | N | N | N | High |
| da Silva Ferreira et al. 2010 [66] | N | N | Y | Y | Y | N | N | High |
| Ural et al. 2010 [46] | N | Y | Y | N | N | N | Y | High |
| Spohr et al. 2008 [76] | N | N | Y | N | N | N | Y | High |
| Shiu et al. 2007 [67] | N | Y | Y | N | N | N | N | High |
| Da Silveira et al. 2005 [75] | N | Y | Y | Y | N | N | Y | Medium |
