# Supplementary figures and images for: The effects of lasers on bond strength to ceramic materials: A systematic review and meta-analysis
Source: PLoS One. 2018 Jan 2;13(1):e0190736. doi: 10.1371/journal.pone.0190736 (PMC5749860; doi:10.1371/journal.pone.0190736)

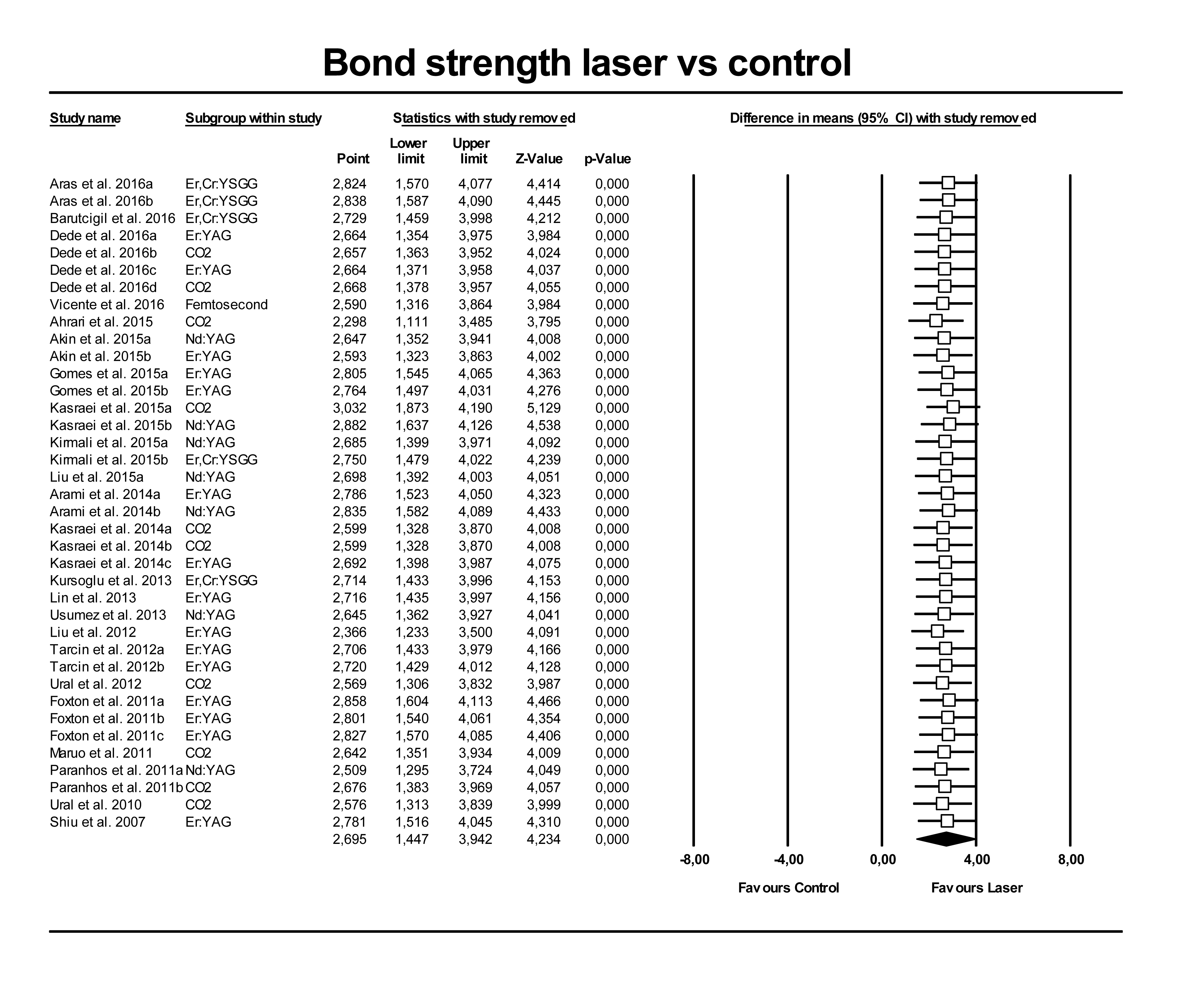

Supplement: S1 Fig — (TIF) [file pone.0190736.s003.tif]

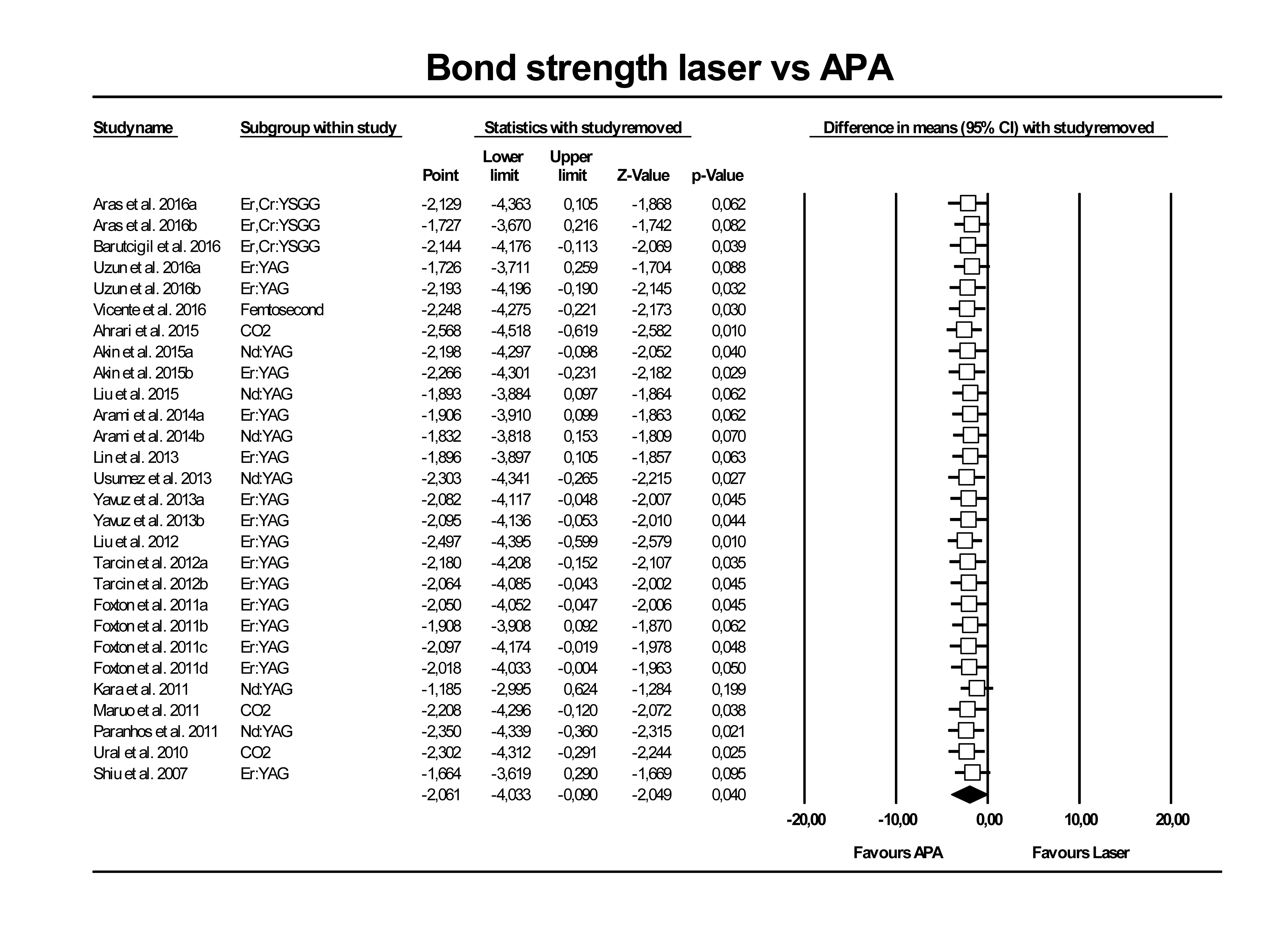

Supplement: S2 Fig — (TIF) [file pone.0190736.s004.tif]
